# GAS6/AXL signaling promotes M2 microglia efferocytosis to alleviate neuroinflammation in sepsis-associated encephalopathy

**DOI:** 10.1038/s41420-025-02507-8

**Published:** 2025-06-06

**Authors:** Yuedong Tang, Hanbing Hu, Qiliang Xie, Jie Shen

**Affiliations:** 1 Center of Emergency and Critical Medicine, Jinshan Hospital of Fudan University, Shanghai, 201508 China; 2 Shanghai Institute of Infectious Disease and Biosecurity, Fudan University, Shanghai, 200032 China; 3 Research Center for Chemical Injury, Emergency and Critical Medicine of Fudan University, Shanghai, 201508 China; 4 Jinshan Hospital of Fudan University, Key Laboratory of Chemical Injury, Emergency and Critical Medicine of Shanghai Municipal Health Commission, Shanghai, 201508 China

**Keywords:** Molecular biology, Diseases

## Abstract

Sepsis-associated encephalopathy (SAE) is a severe complication marked by acute central nervous system (CNS) injury and neuroinflammation. M2 microglia efferocytosis is essential for resolving neuroinflammation, but its regulatory mechanisms remain unclear. This study explored the GAS6/AXL signaling pathway in SAE, hypothesizing its role in enhancing anti-inflammatory responses and efferocytosis. A mouse model of SAE was established via cecal ligation and puncture (CLP), and cognitive impairments were assessed through behavioral tests. Brain tissues and microglia were isolated for RNA sequencing (RNA-Seq) to identify genes associated with the GAS6/AXL pathway. Recombinant GAS6 (rGAS6) protein and an AXL inhibitor were used to examine the pathway’s effects on microglial Rac1 activity and functionality. Results demonstrated that GAS6/AXL activation significantly upregulated anti-inflammatory cytokines, enhanced efferocytosis, and suppressed pro-inflammatory responses, improving cognitive outcomes. These findings highlight GAS6/AXL as a critical modulator of microglial functions, providing a promising molecular target for treating SAE.

**Figure:**
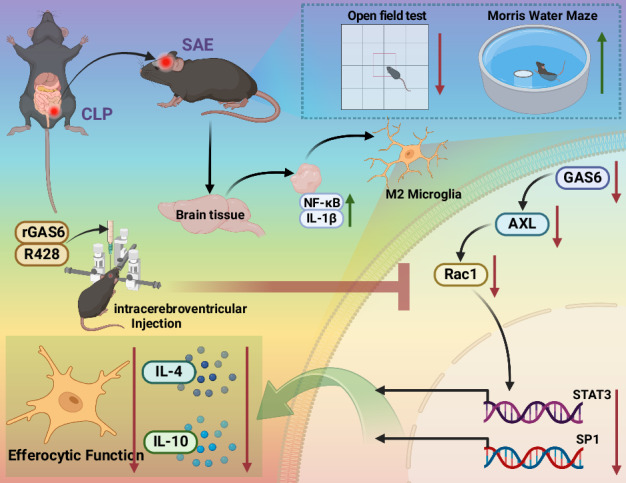
GAS6/AXL Pathway Reduces Neuroinflammation in SAE via Regulation of Anti-Inflammatory and Efferocytic Function in M2 Microglia.

GAS6/AXL Pathway Reduces Neuroinflammation in SAE via Regulation of Anti-Inflammatory and Efferocytic Function in M2 Microglia.

## Introduction

Sepsis-associated encephalopathy (SAE) is a severe complication of sepsis, characterized by acute central nervous system injury and neuroinflammatory responses. This condition often leads to neurological symptoms such as cognitive dysfunction, confusion, and memory impairment [1]. Epidemiological studies indicate that the incidence of SAE is relatively high among sepsis patients and is closely associated with increased mortality rates. The pathological mechanisms involve the release of various cytokines and inflammatory mediators [2], which cross the blood-brain barrier, impair brain function [3], and trigger widespread neuroinflammatory responses [4]. However, the precise molecular mechanisms underlying SAE remain incompletely understood [5], limiting the development of effective therapeutic approaches [6]. Current treatment strategies primarily focus on controlling the underlying cause of sepsis and its systemic inflammatory response, with research on specific neuroprotective therapies still in its early stages. Therefore, understanding SAE’s molecular and cellular mechanisms is crucial for developing targeted neuroprotective strategies.

Microglia, the resident immune cells of the central nervous system, play a crucial role in maintaining neural tissue homeostasis and mediating neuroinflammatory processes. Upon injury signals in the nervous system, microglia become rapidly activated and release inflammatory mediators [4], such as tumor necrosis factor-α (TNF-α) and interleukin-1β (IL-1β), which can exacerbate neuronal damage [7]. Additionally, microglia exhibit polarization behavior, differentiating into pro-inflammatory M1 and anti-inflammatory M2 phenotypes in response to distinct environmental signals [8]. M2 microglia contribute to post-injury repair and inflammation resolution by phagocytosing dead cells and cellular debris, as well as releasing anti-inflammatory factors such as IL-4 and IL-10 [9], thereby mitigating the inflammatory response. Enhancing the function of M2 microglia, particularly efferocytosis, may offer a novel therapeutic strategy for treating neuroinflammation [10]. A large number of studies have shown that M2 microglia exhibit high phagocytic ability in various neuroinflammation and injury models, which is closely related to their alternative activation state. Specifically, classical M2 polarization is usually induced by anti-inflammatory cytokines such as IL-4 and IL-13. These cytokines activate the STAT6 and PPARγ signaling pathways, which not only promote the expression of anti-inflammatory genes but also upregulate many receptors related to phagocytic function, such as CD206 (mannose receptor) and TREM2 [11, 12]. Additionally, M2 microglia possess strong abilities to clear cell debris, dead cells, and pathological protein aggregates, which help alleviate local inflammation and promote tissue repair. Their high phagocytic ability is reflected not only in the expression levels of phagocytic receptors on the cell surface but also in enhanced intracellular signaling and cytoskeletal remodeling [13, 14].

The GAS6/AXL signaling pathway is critical in various biological processes, including cell survival, proliferation, migration, and immune regulation. As the AXL receptor tyrosine kinase ligand, GAS6 activates this pathway and initiates multiple downstream signaling events [15]. This pathway is particularly important for regulating inflammatory responses and promoting cell survival. In models of neurological diseases, activation of GAS6/AXL has been shown to suppress excessive immune responses and facilitate neural tissue repair [16]. Specifically, following brain injury, GAS6/AXL activation promotes neuroprotection and reduces neuronal death. Recent studies also indicate that the GAS6/AXL pathway has potential anti-inflammatory effects by modulating microglial reactivity and function, offering a new perspective for treating neuroinflammatory diseases.

RNA sequencing (RNA-Seq) is a powerful tool for gene expression analysis, enabling researchers to identify genes with altered expression under specific disease states [17] and to uncover the regulatory networks responsible for these changes. In the context of neuropathology, RNA-Seq has been widely used to investigate the molecular mechanisms underlying inflammation, neurodegenerative diseases, and neural injury [18]. By comparing gene expression profiles between diseased and control conditions, RNA-Seq can identify critical biomarkers and potential therapeutic targets [19]. In this study, we used RNA-Seq to analyze gene expression systematically changes in microglia from a mouse model of SAE, focusing on genes related to the GAS6/AXL signaling pathway.

The primary objective of this study was to investigate the molecular mechanisms of the GAS6/AXL signaling pathway in SAE using RNA-Seq technology, focusing on how this pathway regulates the anti-inflammatory and efferocytic function of microglia. Our experiments confirmed that activation of the GAS6/AXL pathway significantly increased the proportion of M2-type microglia, alleviating neuroinflammation in the SAE mouse model by promoting efferocytosis and the expression of anti-inflammatory factors. These findings enhance our understanding of the regulatory mechanisms underlying neuroinflammation in SAE and provide a scientific basis for developing novel therapies targeting the GAS6/AXL pathway. Given the high incidence and mortality associated with SAE, the therapeutic strategy proposed in this study holds promise for providing more effective clinical treatment options, ultimately improving patient prognosis and quality of life.

## Results

### Establishment and behavioral assessment of the SAE mouse model

**Fig. 1:**
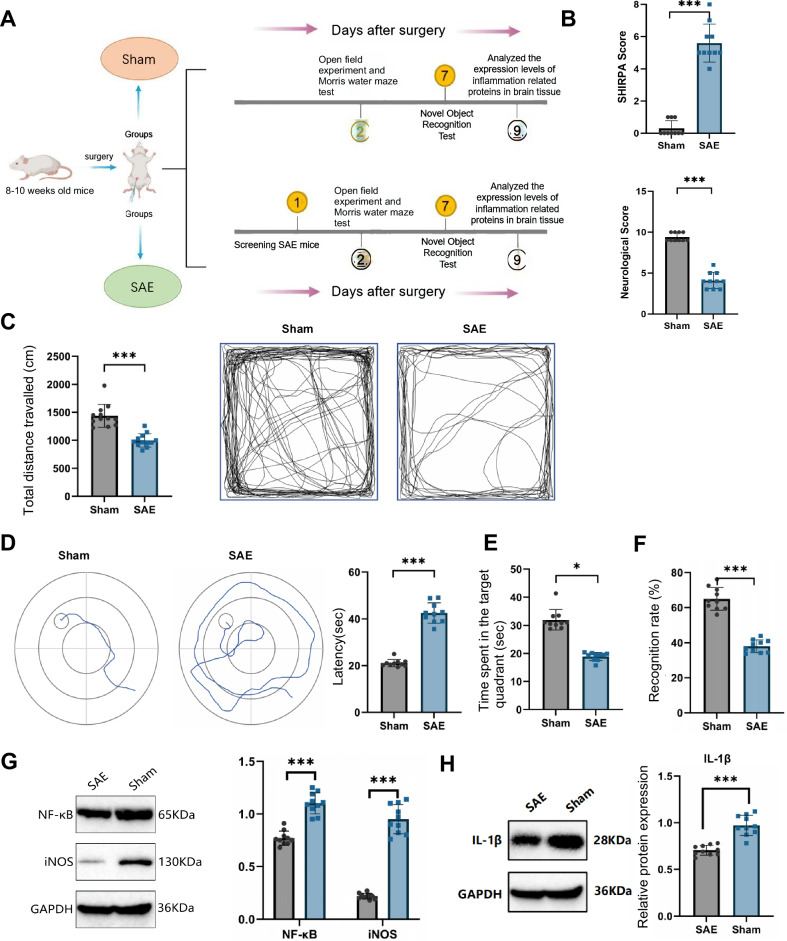
Establishment and behavioral assessment of the SAE mouse model. Note: (**A**) Schematic of the animal experiment process; (**B**) Comparison of SHIRPA and neurological scores between SAE and Sham groups 24 h post-CLP surgery; (**C**) comparison of spontaneous activity distance in the Open Field Test between SAE and Sham groups; (**D**) comparison of escape latency during training sessions in the Morris Water Maze between SAE and Sham groups; (**E**) comparison of time spent in the target quadrant in the Morris Water Maze probe test between SAE and Sham groups; (**F**) comparison of novel object recognition rate between SAE and Sham groups; (**G**) western blot analysis of NF-κB and iNOS expression levels in brain tissue of SAE and Sham groups; (**H**) western blot analysis of IL-1β expression levels in brain tissue of SAE and Sham groups. *N* = 10. Results are presented as mean ± SEM, with statistical significance determined by one-way ANOVA: *p < 0.05, **p < 0.01, ***p < 0.001.

This study successfully established a SAE mouse model using CLP surgery (Fig. 1A). Within 24 h post-surgery, SAE mice were selected based on SHIRPA and neurological scores. Results indicated that SAE model mice had significantly higher SHIRPA scores than those in the Sham group. Additionally, these mice significantly decreased in neurological scores, meeting the SAE model selection criteria (Fig 1B).

Forty-eight hours after establishing the SAE model, open field and Morris water maze tests were conducted to evaluate spontaneous activity and cognitive and memory functions in the mice. In the open field test, SAE mice demonstrated a significant reduction in total distance traveled compared to the Sham group (Fig. 1C). Additionally, their time spent in the central area was significantly reduced, indicating heightened anxiety-like behavior.

The Morris water maze test results showed that the escape latency during the training phase was significantly prolonged in the SAE group compared to the Sham group (Fig. 1D). In the probe test, SAE mice spent significantly less time in the target quadrant, indicating impaired cognitive function (Fig. 1E). A novel object recognition test was conducted following the Morris water maze test to validate cognitive and memory impairments in SAE mice. The results indicated a significantly lower recognition rate for the novel object in the SAE group compared to the Sham group (Fig. 1F). These findings further confirm cognitive and memory deficits in the SAE model mice.

To gain deeper insight into neuroinflammatory responses in the SAE model, we analyzed the expression levels of inflammation-related proteins in brain tissue using Western blot. Results revealed that NF-κB and iNOS expression in the brains of SAE mice were significantly upregulated compared to the control group. (Fig. 1G). Additionally, the expression of pro-inflammatory cytokines such as IL-1β was significantly upregulated in the SAE group compared to the control group (Fig. 1H). These data provide further evidence of marked neuroinflammation in SAE mice.

### Activation of the GAS6/AXL signaling pathway attenuates neuroinflammation in SAE via regulation of Rac1 activity

GAS6 is a 75 kDa vitamin K-dependent protein that participates in immune regulation and inflammation through its interaction with AXL, a member of the TAM receptor family [20].

**Fig. 2:**
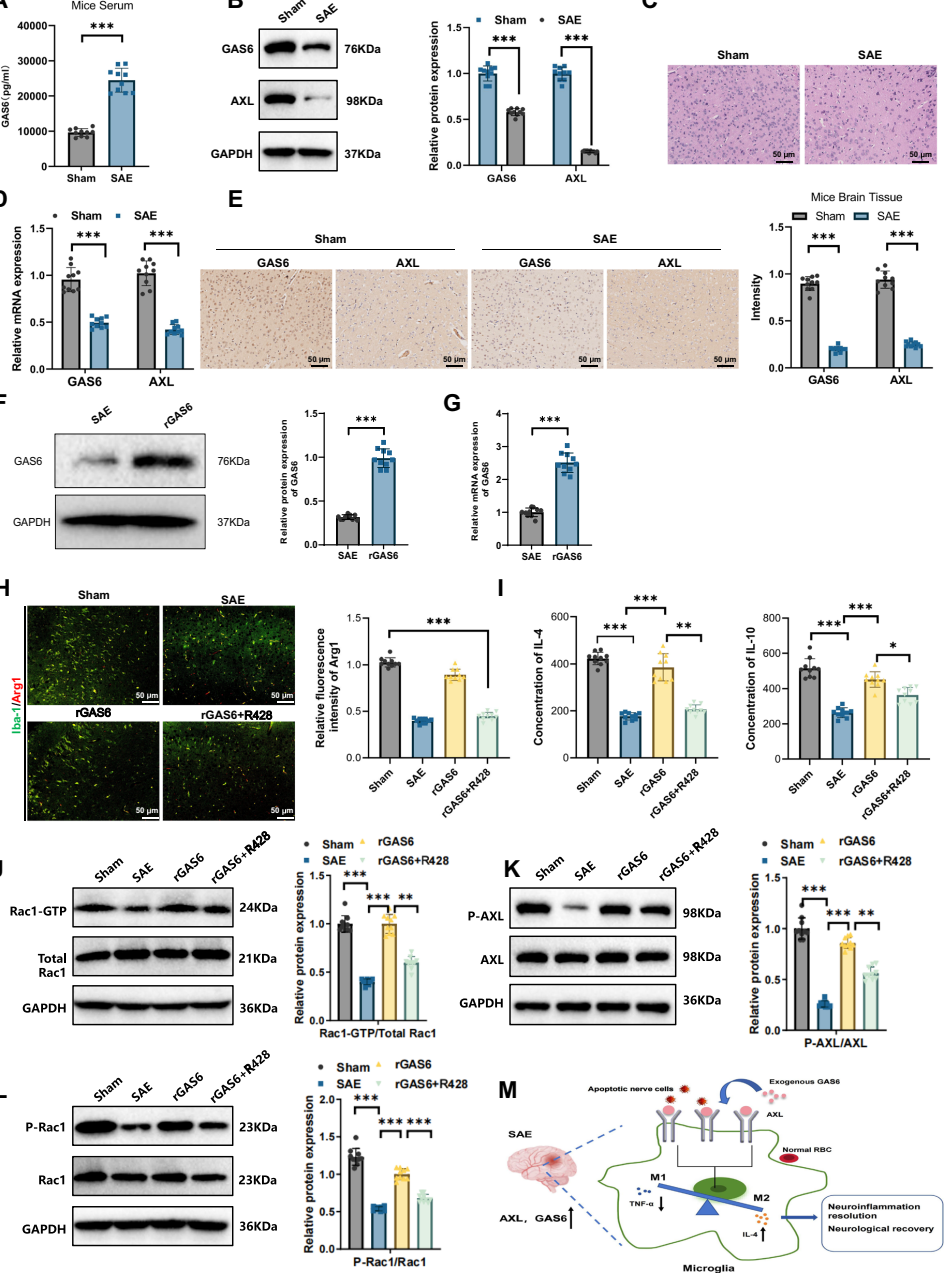
The GAS6/AXL signaling pathway activation mitigates neuroinflammation in SAE by regulating Rac1 activity. Note: (**A**) ELISA analysis of serum GAS6 levels in Sham and SAE groups; (**B**) western blot analysis of serum GAS6 levels in Sham and SAE groups; (**C**) H&E staining of brain tissue to observe histological changes in each group; (**D**) RT-qPCR analysis of relative mRNA expression levels of GAS6 and AXL in each group; (**E**) immunohistochemical analysis of GAS6 and AXL in brain tissue (scale bar: 50 μm); (**F**) western blot analysis of GAS6 in the SAE and rGAS groups; (**G**) RT-qPCR analysis of the relative mRNA expression of GAS6 in each group of mice; (**H**) immunofluorescence staining revealed the proportion of Arg1-positive cells in the brain tissues of mice from different groups; (**I**) ELISA analysis of anti-inflammatory cytokines IL-4 and IL-10 concentrations in brain tissue; (**J**) Rac1 activity assay indicating changes in Rac1-GTP binding in brain tissue across groups; (**K**) western blot analysis of phosphorylated AXL levels in brain tissue of each group; (**L**) western blot analysis of phosphorylated Rac1 levels in brain tissue of each group; (**M**) schematic representation of the GAS6/AXL signaling pathway’s anti-inflammatory and protective roles in microglia via Rac1 activity regulation. *N* = 10. Data are presented as mean ± SEM, with statistical significance determined by one-way ANOVA: *p < 0.05, **p < 0.01, ***p < 0.001.

As shown in Fig. 2A, B, serum GAS6 levels were significantly elevated in SAE mice compared to the Sham group, while brain tissue GAS6 levels were markedly reduced in SAE mice. Hematoxylin and eosin (H&E) staining revealed well-preserved, tightly arranged cellular structures in the brains of Sham mice, whereas SAE mice displayed loosely arranged cells and increased pathological changes (Fig. 2C). Consistent with these findings, RT-qPCR results showed that the relative mRNA expression levels of GAS6 and AXL in SAE mouse brains were significantly lower than those in the Sham group (Fig. 2D). Immunohistochemistry further confirmed reduced staining intensities of GAS6 and AXL in the brains of SAE mice compared to Sham controls (Fig. 2E).

In further investigations, we observed that activation of the GAS6/AXL signaling pathway significantly affected microglial function in SAE mice. For this study, mice were randomly divided into four groups (*n* = 10 per group): Sham, SAE, rGAS6 (treated with rGAS6 via intracerebroventricular injection), and rGAS6 + AXL inhibitor (R428, 125 mg/kg, p.o.). To confirm the efficacy of rGAS6, we measured GAS6 levels in SAE mice treated with rGAS using qPCR and Western blot. The results showed that rGAS injection significantly increased GAS6 protein and mRNA levels in the brain tissue of the mice (Fig. 2F, G).

Immunofluorescence staining revealed the proportion of Arg1-positive cells in the brain tissues of mice from different groups. The results demonstrated a significant reduction in Arg1-positive M2 microglial cells in the SAE group, accompanied by an enhanced inflammatory response. Treatment with rGAS6 effectively restored the proportion of Arg1-positive cells, whereas co-treatment with R428 attenuated the anti-inflammatory effects of rGAS6 (Fig. 2H). These findings further underscore the critical role of Rac1 in microglial function.

Additionally, ELISA results showed that the concentrations of the anti-inflammatory cytokines IL-4 and IL-10 in the brain tissue of SAE mice were significantly reduced compared to the Sham group (Fig. 2I). Concurrently, Rac1 activity assays demonstrated a significant reduction in Rac1-GTP binding in the brains of SAE mice, indicating marked suppression of the Rac1 signaling pathway in these mice (Fig. 2J).

To further confirm the impact of the GAS6/AXL signaling pathway on Rac1 activity, we analyzed the expression and phosphorylation levels of AXL and Rac1 by Western blot. Results indicated that the phosphorylation levels of AXL and Rac1 were significantly reduced in SAE mice, while their total protein levels remained relatively unchanged (Fig. 2K, L). These findings support a mechanism by which the GAS6/AXL signaling pathway modulates microglial function via regulation of Rac1 phosphorylation.

These experimental results demonstrate that the successfully established SAE mouse model exhibits marked behavioral deficits and neuroinflammatory responses. The GAS6/AXL signaling pathway plays an essential anti-inflammatory and protective role in microglia by regulating Rac1 activity (Fig. 2M).

### The GAS6/AXL signaling pathway enhances anti-inflammatory and efferocytic function of M2 microglia by modulating gene expression profiles

**Fig. 3:**
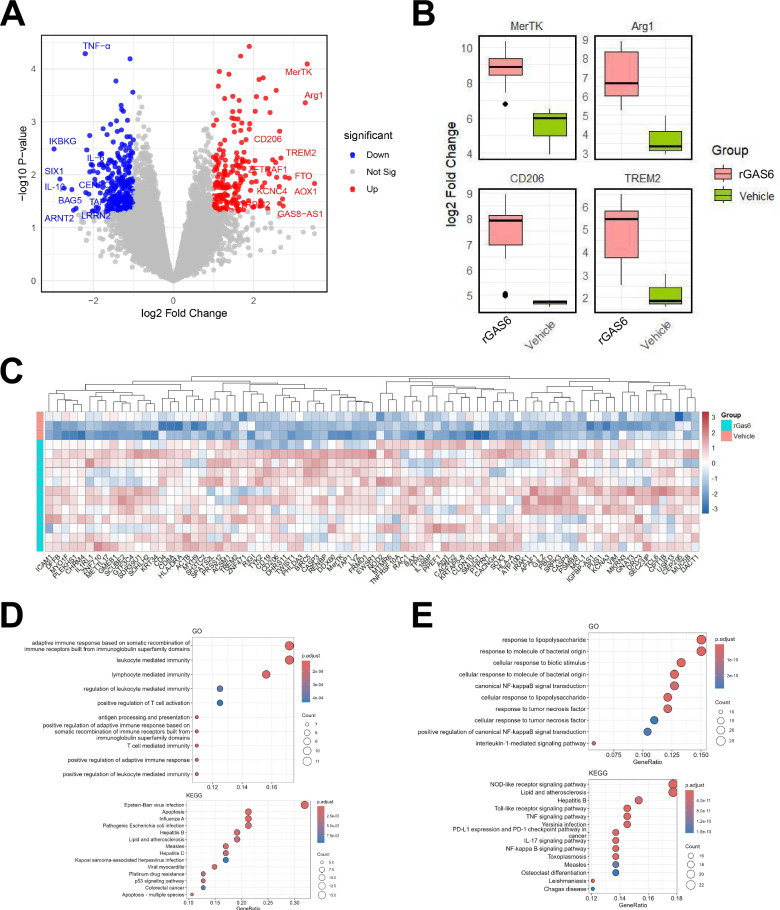
Differential gene expression and functional enrichment analysis of microglia in the rGAS6 and vehicle groups. Note: (**A**) differential gene expression in microglia between the rGAS6-treated and Vehicle groups. rGAS6 treatment significantly altered gene expression in microglia, upregulating anti-inflammatory and efferocytosis-related genes (e.g., Arg1, CD206, MerTK, TREM2) and downregulating pro-inflammatory genes (e.g., TNF-α, IL-1β, IL-6); (**B**) rGAS6 treatment enhanced the expression of genes associated with anti-inflammatory and efferocytic function in microglia, promoting immune regulation and apoptotic cell clearance through the upregulation of key genes (e.g., Arg1, MerTK); (**C**) cluster analysis illustrating distinct gene expression profiles in microglia between the rGAS6-treated and Vehicle groups; (**D**) GO and KEGG enrichment analyses showing significant enrichment of upregulated genes in pathways related to immune response, efferocytosis, and anti-inflammatory activity; (**E**) GO and KEGG enrichment analyses showing significant enrichment of downregulated genes in pathways associated with NF-κB signaling and pro-inflammatory cytokine signaling. *n* = 3.

Differential gene expression analysis of RNA-seq data was performed using the DESeq2 package. Results showed that compared to the Vehicle group, a total of 450 genes were significantly differentially expressed in microglia from the rGAS6-treated group (log_2_FC > 1, *p* < 0.05), including 252 downregulated genes and 198 upregulated genes (Fig. 3A). Notably, genes such as MerTK, Arg1, CD206, and TREM2 were significantly upregulated in the rGAS6-treated group. MerTK and TREM2, closely related to efferocytosis function, likely enhance the microglia’s ability to clear apoptotic cells, thereby and promoting tissue repair and maintaining tissue homeostasis.

Arg1, an important marker of M2 macrophage polarization, was highly expressed, suggesting a shift in microglia towards an anti-inflammatory role. CD206, a classical marker of M2 microglia, further validated that rGAS6 treatment induced microglial polarization toward the anti-inflammatory M2 phenotype. These findings suggest that the GAS6/AXL signaling pathway may regulate microglial anti-inflammatory and efferocytosis function by upregulating these genes (Fig. 3B).

To further understand the global expression patterns of differentially expressed genes, we performed cluster analysis on significantly upregulated genes. Results indicated that immune response-related genes, including ICAM1, CD109, and HLA-DRA, were significantly upregulated following rGAS6 treatment. Genes related to cell motility, such as ACTG1, S100A6, and MYL9, showed notable upregulation. Cell survival genes such as BIRC5, NOD2, and CASP3 were also significantly elevated in the rGAS6-treated group. Non-apoptotic CASP3 activity can enhance microglial efferocytic function, improving their ability to clear apoptotic cells and damaged tissues, promoting tissue repair and homeostasis maintenance [21]. Notably, genes associated with efferocytosis function, including MerTK and TREM2, were upregulated. MerTK and TREM2 are key factors in the microglial clearance of apoptotic cells and damaged tissue, suggesting that rGAS6 treatment enhanced the microglial capacity for clearance and repair. The observed changes in gene expression indicate that the GAS6/AXL signaling pathway may play a crucial role in enhancing microglial anti-inflammatory, efferocytosis function, and cell survival functions by regulating genes associated with immune response, cell motility, cell survival, and efferocytosis (Fig. 3C). This gene expression pattern suggests that activation of the GAS6/AXL pathway broadly modulates microglial function, particularly in enhancing anti-inflammatory and efferocytosis activities.

Next, we conducted GO and KEGG functional enrichment analyses, which revealed that the upregulated genes were significantly enriched in pathways related to immune response, efferocytosis, and anti-inflammatory processes, such as “phagosome,” “PI3K-Akt signaling pathway,” and “macrophage polarization” (Fig. 3D). Efferocytosis is a crucial clearance mechanism for macrophages and microglia, especially for maintaining tissue homeostasis and reducing inflammatory responses by clearing apoptotic cells.

Previous studies have demonstrated that the GAS6/AXL signaling pathway is essential in efferocytosis function, particularly by enhancing macrophage engulfment of apoptotic cells and thus improving the immune system’s clearance capacity [22]. Additionally, research indicates that the GAS6/AXL pathway enhances cell survival and anti-inflammatory responses via the PI3K-Akt pathway, thereby playing a critical role in modulating microglial functions in the nervous system [23]. This enhancement of efferocytosis function removes damaged or apoptotic cells and suppresses the expression of pro-inflammatory cytokines such as TNF-α, IL-1β, and IL-6, thereby reducing inflammation. Conversely, downregulated genes were enriched in pathways related to the “NF-κB signaling pathway” and “pro-inflammatory cytokine signaling” (Fig. 3E).

These findings further support the crucial role of the GAS6/AXL signaling pathway in regulating microglia’s anti-inflammatory and efferocytosis function. This pathway likely attenuates neuroinflammatory responses by modulating specific signaling pathways, thereby enhancing microglia’s anti-inflammatory and efferocytosis capacity, which has significant neuroprotective effects.

### STAT3 and SP1 regulate M2 microglial functions through the GAS6/AXL signaling pathway, enhancing anti-inflammatory and efferocytosis activity

**Fig. 4:**
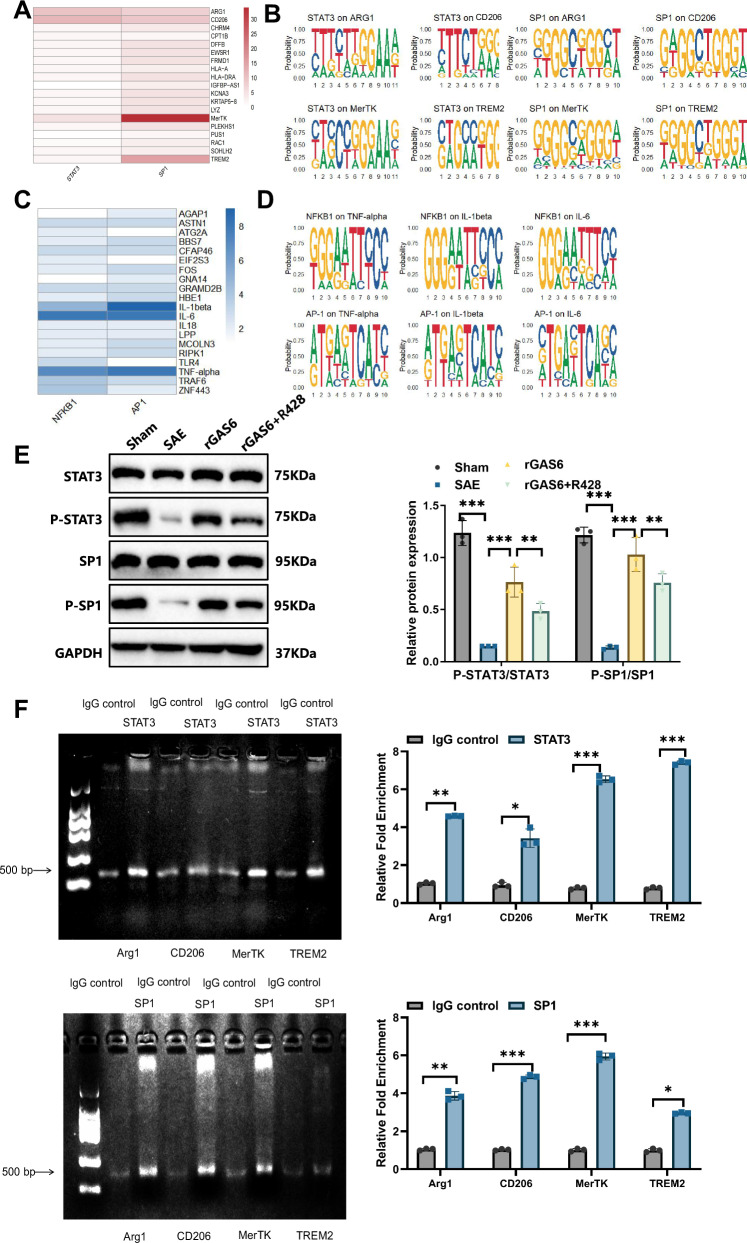
Molecular mechanism analysis of GAS6/AXL pathway-mediated activation of anti-inflammatory and efferocytic function in microglia through STAT3 and SP1. Note: (**A**) JASPAR database predictions indicate significant enrichment of STAT3 and SP1 binding sites in upregulated genes (e.g., Arg1, CD206, MerTK, and TREM2) in the rGAS6-treated microglia; (**B**) sequence logo for STAT3 and SP1 binding motifs within significantly upregulated genes; (**C**) JASPAR database predictions reveal significant enrichment of NF-κB and AP-1 binding sites in downregulated genes (e.g., TNF-α, IL-1β, and IL-6); (**D**) sequence logo for NF-κB and AP-1 binding motifs within significantly downregulated genes; (**E**) western blot analysis of STAT3 and SP1 phosphorylation levels across different treatment groups; (**F**) ChIP analysis of STAT3 and SP1 binding to promoter regions of target genes (Arg1, CD206, MerTK, and TREM2) in rGAS6-treated microglia. All cell experiments were repeated three times, *n* = 3. Results are presented as mean ± SEM, with statistical significance determined by one-way ANOVA: *p < 0.05, **p < 0.01, ***p < 0.001.

We performed transcription factor binding site prediction analysis on differentially expressed genes to further investigate the mechanisms by which the GAS6/AXL signaling pathway regulates M2 microglial function. Bioinformatic analysis using the JASPAR database revealed significant enrichment of STAT3 and SP1 binding sites among genes related to anti-inflammatory and efferocytosis function upregulated in the rGAS6-treated group. This suggests that STAT3 and SP1 play critical roles in modulating the expression of these genes. Specifically, STAT3 and SP1 binding sites were enriched in M2 polarization markers, such as Arg1 and CD206, and in efferocytosis-related genes, including MerTK and TREM2. These findings imply that STAT3 and SP1 may be key regulators in microglia’s GAS6/AXL pathway-mediated anti-inflammatory and efferocytosis function(Fig. 4A).

To further clarify the roles of these transcription factors in regulating M2 microglial function, we analyzed the binding site sequences of STAT3 and SP1 on genes such as ARG1, CD206, MerTK, and TREM2. The binding patterns of STAT3 and SP1 appear to determine the activation of these genes (Fig. 4B). Additionally, NF-κB and AP-1 binding sites were significantly enriched among the downregulated genes, particularly in the regulatory regions of pro-inflammatory cytokines, including TNF-α, IL-1β, and IL-6. This suggests that the GAS6/AXL signaling pathway may mitigate inflammatory responses by inhibiting the activity of these transcription factors, thereby reducing pro-inflammatory gene expression (Fig. 4C).

Binding site analysis on TNF-α, IL-1β, and IL-6 genes showed that NF-κB and AP-1 regulate inflammatory gene expression by interacting with target genes. The significant enrichment of these binding sites supports the hypothesis that NF-κB and AP-1 are inhibited within the GAS6/AXL pathway, reducing pro-inflammatory responses and further enhancing the anti-inflammatory function of microglia (Fig. 4D).

In summary, the GAS6/AXL signaling pathway enhances microglia’s anti-inflammatory and efferocytosis function by activating STAT3 and SP1 while concurrently inhibiting NF-κB and AP-1 to reduce pro-inflammatory gene expression and attenuate inflammation. These findings provide new insights into the regulatory mechanisms of the GAS6/AXL pathway in neuroinflammation.

We conducted additional experiments to validate these transcription factors’ roles within the GAS6/AXL pathway. First, we analyzed the phosphorylation levels of STAT3 and SP1 across different treatment groups using Western blot. Results showed that rGAS6 treatment significantly increased the phosphorylation levels of STAT3 (p-STAT3) and SP1 (p-SP1) compared to the control group (Fig. 4E). In the rGAS6 + R428 group, these phosphorylation levels were significantly reduced, returning to levels close to those in the SAE group, further confirming that the GAS6/AXL pathway regulates microglial function via STAT3 and SP1 activation.

We conducted ChIP assays to confirm the direct regulatory effects of STAT3 and SP1 on target genes. Using antibodies specific to STAT3 and SP1, ChIP analysis was performed on microglia in the rGAS6-treated group, followed by qPCR to assess binding at the promoter regions of target genes such as Arg1, CD206, MerTK, and TREM2. Results indicated that STAT3 and SP1 binding at these promoter regions was significantly enhanced in the rGAS6-treated group (Fig. 4F). These findings strongly support that STAT3 and SP1 act as key downstream transcription factors of the GAS6/AXL signaling pathway, directly binding to target gene promoters to promote the anti-inflammatory and efferocytic function of M2 microglia.

**Fig. 5:**
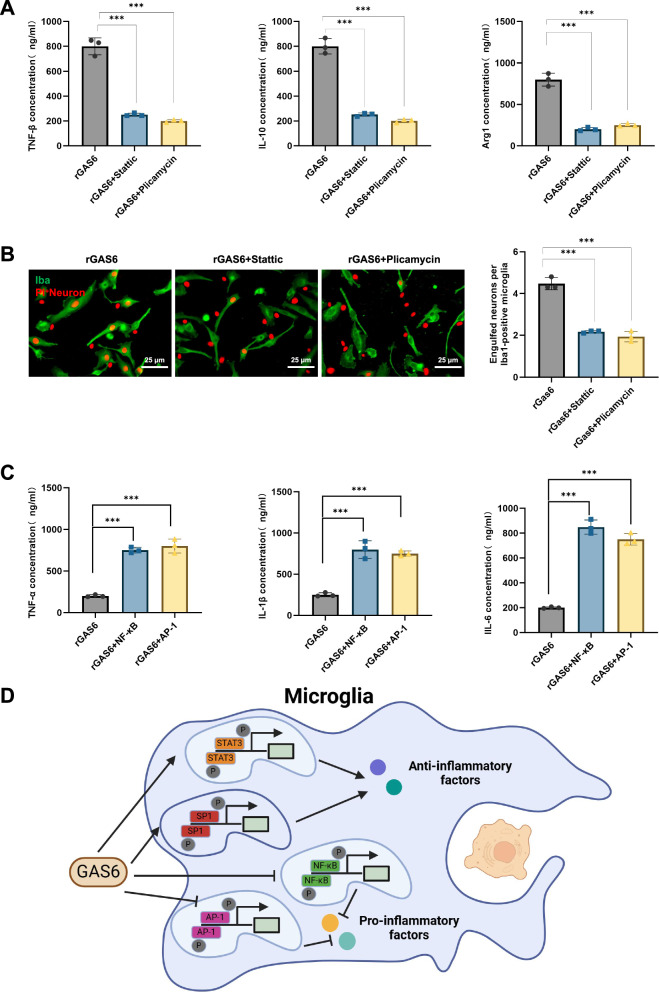
Molecular mechanism analysis of GAS6/AXL pathway activation of anti-inflammatory and efferocytic functions in microglia via STAT3 and SP1. Note: (**A**) ELISA results showing secretion levels of anti-inflammatory factors TNF-β, IL-10, and Arg1 in rGAS6-induced microglia following treatment with specific STAT3 and SP1 inhibitors, and expression levels of pro-inflammatory factors TNF-α, IL-1β, and IL-6 in rGAS6-induced microglia following NF-κB and AP-1 inhibition; (**B**) fluorescence microscopy analysis demonstrating efferocytic capacity of rGAS6-induced microglia for PI-labeled neurons following treatment with STAT3 and SP1 inhibitors; (**C**) ELISA analysis of pro-inflammatory cytokine secretion, specifically TNF-α, IL-1β, and IL-6; (**D**) schematic diagram illustrating GAS6/AXL pathway activation via STAT3 and SP1, enhancing anti-inflammatory and efferocytic responses in M2-polarized microglia, alongside NF-κB and AP-1 inhibition reducing pro-inflammatory gene expression. All cell experiments were performed in triplicate, *n* = 3. Results are presented as mean ± SEM, with statistical significance determined by one-way ANOVA: ***p < 0.001.

Finally, to further confirm the functional roles of STAT3 and SP1 within the GAS6/AXL signaling pathway, we treated microglia with the specific STAT3 inhibitor Stattic and the SP1 inhibitor Plicamycin. Results showed that inhibiting STAT3 and SP1 activity significantly reduced the rGAS6-induced secretion of anti-inflammatory factors TNF-β, IL-10, and Arg1 compared to the control group (Fig. 5A). The efferocytic function also declined markedly, with the phagocytosis of PI-labeled neurons significantly reduced compared to the control group (Fig. 5B). Furthermore, enhancing NF-κB and AP-1 expression in rGAS6-treated microglia significantly increased the secretion of pro-inflammatory cytokines TNF-α, IL-1β, and IL-6 (Fig. 5C). These findings confirm that STAT3 and SP1 are critical downstream effectors of the GAS6/AXL pathway, playing essential roles in regulating microglial function.

In summary, the transcription factors STAT3 and SP1, upon activation by the GAS6/AXL signaling pathway, significantly enhance the anti-inflammatory and efferocytic function of M2 microglia. Conversely, inhibiting NF-κB and AP-1 activity may be a key mechanism for downregulating pro-inflammatory gene expression (Fig. 5D). These findings further elucidate the molecular basis of the neuroprotective effects of the GAS6/AXL pathway in SAE.

### The GAS6/AXL signaling pathway mediates anti-inflammatory responses in microglia by regulating cytokine expression

**Fig. 6:**
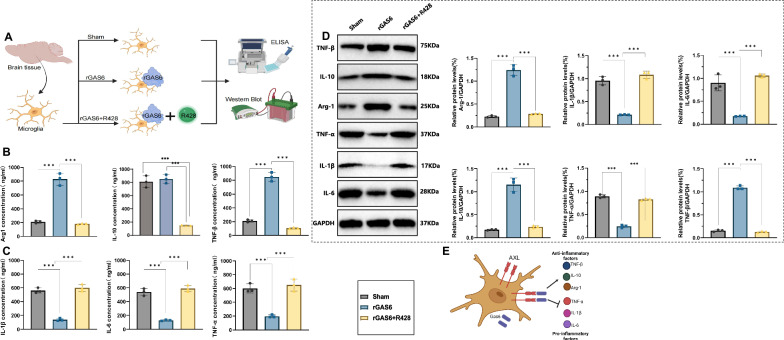
Mechanism analysis of GAS6/AXL pathway regulation of inflammatory response in microglia. Note: (**A**) flowchart of the cell experiment; (**B**) ELISA analysis of anti-inflammatory factor secretion levels (TNF-β, IL-10, and Arg1) across experimental groups (Sham, rGAS6, and rGAS6 + R428 groups); (**C**) ELISA analysis of pro-inflammatory cytokine secretion levels (TNF-α, IL-1β, and IL-6) across experimental groups; (**D**) western blot analysis of protein expression levels for TNF-β, IL-10, Arg1, TNF-α, IL-1β, and IL-6 across groups; (**E**) schematic illustrating the mechanism by which the GAS6/AXL pathway modulates the inflammatory state in microglia. All experiments were performed in triplicate (*n* = 3). Data are presented as mean ± SEM, with statistical significance determined by one-way ANOVA: ***p < 0.001.

Next, microglia were divided into three experimental groups: Sham, rGAS6, and rGAS6 + R428. Comparing these groups allowed us to elucidate the role of the GAS6/AXL pathway in modulating microglial inflammation and efferocytic function (Fig. 6A).

First, ELISA assays were conducted to measure cytokine levels in cerebrospinal fluid. Results showed that rGAS6 treatment significantly increased the secretion of anti-inflammatory factors TNF-β, IL-10, and Arg1 compared to the Sham group, while these effects were notably suppressed in the rGAS6 + R428 group (Fig. 6B). In contrast, pro-inflammatory cytokines TNF-α, IL-1β, and IL-6 were significantly reduced in the rGAS6-treated group, with levels restored in the rGas6 + R428 group (Fig. 6C). Additionally, Western blot analysis of anti-inflammatory factors TNF-β, IL-10, and Arg1, as well as pro-inflammatory cytokines TNF-α, IL-1β, and IL-6, confirmed similar trends across groups (Fig. 6D).

These results indicate that the GAS6/AXL signaling pathway mediates anti-inflammatory responses in microglia by modulating the expression of inflammatory cytokines (Fig. 6E).

### Activation of the GAS6/AXL signaling pathway enhances efferocytic function through Rac1

Rac1, a member of the Rho family of small GTPases, plays a crucial role in efferocytic function, and its signaling pathway is closely associated with efferocytosis [24].

**Fig. 7:**
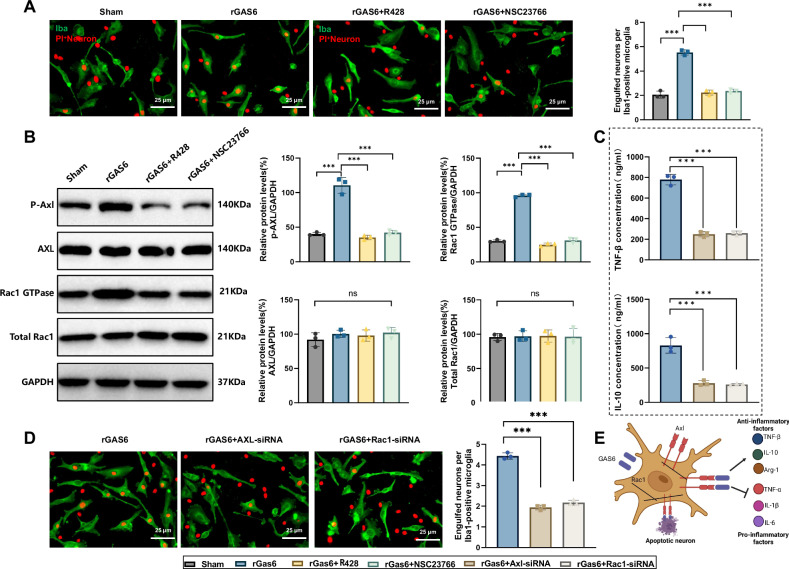
GAS6/AXL enhances anti-inflammatory and efferocytic function in microglia via the Rac1 signaling pathway. Note: (**A**) fluorescence microscopy analysis showing phagocytosis of PI-labeled neurons by microglia across experimental groups (Sham, rGAS6, rGAS6 + R428, and rGAS6 + NSC23766); (**B**) western blot analysis of AXL and Rac1 phosphorylation levels and Rac1 GTP-binding activity across groups; (**C**) secretion levels of anti-inflammatory factors TNF-β and IL-10 in microglia after AXL and Rac1 knockdown via siRNA; (**D**) efferocytic capacity of microglia after AXL and Rac1 knockdown via siRNA; (**E**) schematic overview of the GAS6/AXL pathway’s regulation of anti-inflammatory and efferocytic function in microglia via the Rac1 pathway. All experiments were conducted in triplicate (*n* = 3). Results are presented as mean ± SEM, with statistical significance determined by one-way ANOVA: ***p < 0.001.

To evaluate the impact of the GAS6/AXL pathway on microglial efferocytic function, we established the following experimental groups: Sham, rGAS6, rGAS6 + R428, and rGas6 + NSC23766. Using fluorescence microscopy, we analyzed the ability of microglia to engulf PI-labeled neurons. Results showed that, compared to the Sham group, the efferocytosis capacity of microglia in the rGAS6-treated group was significantly enhanced. However, this efferocytic ability was markedly reduced in the rGAS6 + R428 group, and inhibition of Rac1 activity with NSC23766 in the rGAS6 + NSC23766 group similarly attenuated rGAS6-induced efferocytosis (Fig. 7A). These findings further confirm the critical role of the GAS6/AXL pathway in regulating microglial efferocytic function, particularly through mediation by the Rac1 pathway.

To further validate the roles of AXL and Rac1 in the GAS6 signaling pathway, we analyzed the expression and phosphorylation levels of AXL and Rac1 across different treatment groups using Western blot. Results indicated that rGAS6 treatment significantly increased AXL phosphorylation and enhanced Rac1 GTP-binding activity. In both the rGAS6 + R428 and rGas6 + NSC23766 groups, these elevated phosphorylation levels and Rac1 activity were notably reduced (Fig. 7B), further supporting that the GAS6/AXL pathway regulates microglial function through Rac1 activation.

To confirm the specific functions of AXL and Rac1 within the GAS6 pathway, we employed siRNA to knock down AXL and Rac1 genes. Results showed that the knockdown of AXL or Rac1 significantly diminished rGAS6-induced anti-inflammatory cytokine secretion and efferocytic capacity in microglia (Fig. 7C, D). These findings clearly indicate that AXL and Rac1 play critical roles in mediating microglia’s anti-inflammatory and efferocytic function within the GAS6/AXL pathway.

These results suggest that activation of the GAS6/AXL signaling pathway promotes anti-inflammatory responses and enhances efferocytic function in microglia via the Rac1 pathway, which is crucial for alleviating neuroinflammatory responses in SAE (Fig. 7E).

## Discussion

SAE is a severe complication of sepsis, characterized by extensive central nervous system damage and neuroinflammatory responses [7, 25]. SAE profoundly impacts patients’ cognitive function and quality of life [26], yet effective treatment options remain limited due to its complex pathophysiology [1, 27]. Despite recent studies focusing on the neuropathological underpinnings of sepsis, the molecular mechanisms underlying are SAE still not fully understood [28]. This study utilizes advanced RNA-Seq technology to explore the regulatory role of the GAS6/AXL signaling pathway in an SAE mouse model, aiming to provide new insights into the molecular pathophysiology of SAE and identify potential therapeutic targets.

Our findings indicate that activation of the GAS6/AXL signaling pathway significantly enhances the efferocytic function of M2 microglia, consistent with previous studies showing that GAS6/AXL signaling modulates inflammation by promoting M2 immune cell function in various inflammatory models [29, 30]. However, our study offers a novel perspective by focusing specifically on how this pathway mitigates neuroinflammation in the SAE model by regulating microglial efferocytic capacity, a mechanism that has yet to be fully explored in previous research. Additionally, we observed increased Rac1 activity, which has rarely been directly linked to the GAS6/AXL pathway in existing literature, suggesting the potentially discovery of a new signaling mechanism in our study.

In investigating the molecular mechanisms underlying anti-inflammatory and efferocytic function, this study employed detailed gene expression analysis and protein validation to demonstrate that rGAS6 treatment increased the proportion of M2 microglia and promoted the secretion of anti-inflammatory factors IL-4 and IL-10. These findings align with previous research on the regulatory effects of GAS6 on anti-inflammatory factors in the broader environment; however, our study further demonstrates that the activation of key molecules such as MerTK and TREM2 mediates this effect. Additionally, our study provides new evidence supporting the roles of downstream transcription factors STAT3 and SP1, which are crucial in regulating anti-inflammatory and efferocytosis-related genes—an aspect rarely discussed in prior literature.

Behavioral results in this study show that SAE mice treated with rGAS6 exhibited significant improvements in the open field test and Morris water maze, suggesting a close association between activation of the GAS6/AXL pathway and neuroprotection, as well as cognitive recovery. Unlike previous studies, our research highlights behavioral improvements and directly links these enhancements to improved microglial function, offering new insights into these behavioral changes’ cellular and molecular basis. These findings have significant implications for developing novel therapeutic strategies, particularly in improving neurological function in SAE patients by targeting specific signaling pathways.

This study also explored the potential interactions between the GAS6/AXL signaling pathway and other inflammation-regulating pathways, particularly its relationship with the NF-κB pathway. Comparisons with previous studies suggest that the GAS6/AXL pathway may interact with the NF-κB pathway through multiple mechanisms to modulate inflammatory responses jointly. While the specific mechanisms of this interaction require further investigation, existing data indicate that understanding these complex signaling networks is essential for developing more effective anti-inflammatory therapies.

This research provides clear molecular evidence for the neuroprotective role of the GAS6/AXL pathway in SAE, highlighting its central role in modulating microglial anti-inflammatory and efferocytic function. These findings deepen our understanding of neuroinflammation, particularly in the complex inflammatory responses triggered by sepsis, and reveal the regulatory potential of the GAS6/AXL pathway in this context. Clinically, GAS6/AXL pathway activators or related drugs could offer a novel therapeutic strategy for treating SAE by enhancing microglia’s anti-inflammatory and clearance capabilities, potentially improving neurological outcomes in sepsis patients. This study thus identifies promising therapeutic targets for the precision treatment of SAE, underscoring significant translational potential.

Although this study reveals the critical role of the GAS6/AXL signaling pathway in SAE, several limitations remain. First, the research focuses on microglia without comprehensively examining other immune cells involved in SAE. During brain tissue collection, we did not differentiate between the hippocampus and cortical regions, so we cannot compare the two regions. Performing differential analysis of different brain regions to identify the most affected areas in SAE will be a focus of our future research. Second, the mouse model may only partially replicate the complex pathology of human SAE, presenting potential species-specific differences. Additionally, while this study confirms the essential role of the GAS6/AXL pathway in microglia, its behavior in various sepsis models and human clinical samples requires further validation.

Future studies should incorporate clinical patient samples to explore the regulatory effects of the GAS6/AXL signaling pathway in different sepsis conditions and conduct relevant clinical trials to assess its therapeutic potential. Expanding research to include other immune cells and signaling pathways will offer a more comprehensive understanding of SAE pathology and may reveal opportunities for multi-target combination therapies, advancing the development of effective SAE treatment strategies.

## Conclusion

This study systematically reveals the critical role of the GAS6/AXL signaling pathway in SAE through RNA-Seq analysis, demonstrating that activation of this pathway significantly enhances the anti-inflammatory and efferocytic function of M2 microglia. Results indicate that the GAS6/AXL pathway regulates Rac1 activity and the expression of transcription factors STAT3 and SP1, which augment apoptotic cells’ microglial clearance and reduce pro-inflammatory cytokine release. These effects effectively mitigate neuroinflammation and cognitive impairment in SAE mice. This research not only elucidates the mechanistic role of the GAS6/AXL pathway in modulating microglial function but also provides a new perspective on the molecular pathology of SAE.

## Materials and methods

### Construction of the SAE mouse model

To effectively establish a mouse model of SAE, mice were first anesthetized with avertin (50 mg/kg, intraperitoneal injection, MeilunBio,China) before cecal ligation and puncture (CLP) surgery, ensuring appropriate anesthesia throughout the procedure. Sepsis was induced by ligating the cecum and creating a puncture. Twenty-four hours post-surgery, mice underwent SHIRPA scoring and neurological assessment to evaluate the success of the model establishment. The inclusion criteria for the model were a SHIRPA score of ≥3 and a neurological score of ≤6 within 24 h post-surgery [31, 32].

### Intracerebroventricular Injection

Between 24 and 48 h post-CLP surgery, intracerebroventricular injections were administered. The first intracerebroventricular injection was performed 24 h after the CLP surgery, when the neuroinflammatory response had just begun but was still in an interventionable stage. A supplemental injection was given 48 h after the CLP to ensure sustained efficacy. Using a Hamilton syringe (10 µL, Hamilton Company, USA), drugs were injected into the ventricle for different experimental groups: 10% DMSO (control group, Beyotime, China), recombinant GAS6 (rGAS6, 100 ng, Sangon Biotech (Shanghai), China, to activate the GAS6/AXL signaling pathway), Axl-siRNA (10 µg, Invitrogen, USA, to inhibit AXL expression). Injection sites were precisely located according to the Paxinos and Franklin brain atlas to ensure accurate delivery. The injection volume was set at 5 µL with an injection rate of 1 µL/min to allow for slow, even distribution of the drug. Post-injection, the mice were monitored for recovery and behavioral changes to ensure the safety and efficacy of the experimental conditions.

### Behavioral testing

Forty-eight hours after establishing the SAE model, behavioral tests were conducted to assess cognitive and memory functions in the mice. First, the open field test was performed by placing each mouse in a 50 cm × 50 cm open field box, with a video tracking system (EthoVision XT 14.0, Noldus Information Technology, Netherlands) used to record the activity path, total distance traveled, and time spent in the central area over 5 min. Next, the Morris water maze test was conducted in a circular pool with a diameter of 120 cm, and the water temperature was maintained at 22 ± 1 °C. The training phase lasted for five days, with four trials per day, during which the time to find the hidden platform (escape latency) and path length were recorded. The platform was removed for the probe trial on the ninth day, and the time and path spent in the target quadrant were recorded. A novel object recognition test was conducted further to evaluate cognitive and memory functions in the SAE mice.

The experiment used 8- to 10-week-old male C57BL/6 N mice (purchased from Home-SPF (Beijing) Biotechnology Co., Ltd., China), with 10 mice in each group. All mice were housed under a 12-h light/dark cycle at a temperature of 22 ± 1 °C, with access to standard laboratory feed and water. Following the Morris water maze test, the mice underwent adaptive training in the same laboratory environment. An open field box (50 cm × 50 cm × 50 cm, Jiangsu Jiyuan Biotechnology, China) was used for this experiment. Each mouse was first placed in the box for a 10-min acclimation period. During the training phase, two identical objects (cylindrical plastic toys, 10 cm in height and 3 cm in diameter) were positioned at opposite corners of the box, allowing the mice to explore for 5 min. In the testing phase, one of the objects was replaced with a novel object of similar shape but different appearance (a cubic plastic toy, 4 cm in edge length). The mice were then reintroduced to the box for another 5-min exploration period. A video recording system (SMART v3.0, Panlab, Spain) was used to track the mice’s behavior, precisely measuring the time spent exploring the new object. The recognition index, calculated as the percentage of time spent exploring the new object relative to the total exploration time, was used to evaluate object recognition.

### Immunofluorescence staining

Immunofluorescence staining was performed to assess the activation state of M2 microglia. Brain tissue sections (20 µm thick) were fixed with 4% paraformaldehyde (Sigma-Aldrich, USA) for 10 min, followed by permeabilization with 0.3% Triton X-100 (Sigma-Aldrich, USA) for 10 min. Sections were blocked with 5% bovine serum albumin (BSA, Sigma-Aldrich, USA) for 1 h. Subsequently, primary antibodies Iba-1 (1:500, Wako Pure Chemical Industries, Japan) and Arg1 (1:200, Abcam, UK) were applied, and the sections were incubated overnight at 4 °C. The next day, sections were incubated with Alexa Fluor 488-conjugated secondary antibody (1:1000, Invitrogen, USA) and Alexa Fluor 594-conjugated secondary antibody (1:1000, Invitrogen, USA) for 1 h. Images were captured with a confocal fluorescence microscope (Leica SP8, Germany), and ImageJ software (v1.52, National Institutes of Health, USA) was used to analyze the images and calculate the proportion of M2 microglia.

### Enzyme-linked immunosorbent assay (ELISA) assay

ELISA was performed to quantify the levels of inflammatory cytokines IL-4 and IL-10 in brain tissue. First, the brain tissue was homogenized in RIPA lysis buffer (Thermo Fisher Scientific, USA) and supplemented with protease and phosphatase inhibitors (Roche, Switzerland), followed by incubation at 4 °C for 30 min. The lysate was then centrifuged at 12,000 × *g* for 10 min, and the supernatant was collected. Protein concentrations were determined with a BCA Protein Assay Kit (Thermo Fisher Scientific, USA). For each sample, 50 µg of protein was incubated in a 96-well plate, and IL-4 and IL-10 ELISA kits (R&D Systems, USA) were used according to the manufacturer’s instructions. Absorbance was measured at 450 nm with a microplate reader (BioTek, USA), and cytokine concentrations were calculated using a standard curve.

### Rac1 activity assay

To assess Rac1 activation, a pull-down assay was conducted using a Rac1 Activation Assay Kit (Cytoskeleton, USA). Brain tissue or microglia homogenates were prepared, and GST-Pak1-PBD was used to bind Rac1-GTP. The bound Rac1 was detected by Western blot. Homogenized samples were incubated with GST-Pak1-PBD agarose beads with gentle rotation for 30 min. Unbound proteins were removed by centrifugation, and the beads were washed before eluting the bound proteins. Immunoblotting was performed using an anti-Rac1 antibody (1:1000, Cytoskeleton, USA) and HRP-conjugated anti-mouse secondary antibody (1:5000, Sigma-Aldrich, USA). Signals were detected by chemiluminescence (ECL, Thermo Fisher Scientific, USA), and quantification was performed using ImageJ software.

### Western blot analysis

Total protein was extracted from mouse brain tissue using RIPA lysis buffer (Thermo Fisher Scientific, USA), with added protease and phosphatase inhibitors (Roche, Switzerland). The tissue homogenates were incubated at 4 °C for 30 min and then centrifuged at 12,000 × *g* for 10 min to collect the supernatant. Protein concentration was determined using a BCA Protein Assay Kit (Thermo Fisher Scientific, USA). Samples were mixed with 5× SDS loading buffer and heated at 95 °C for 5 min to denature the proteins. Equal amounts of protein (40 µg) were loaded onto a 10% SDS-PAGE gel for electrophoretic separation, then transferred onto a PVDF membrane (Millipore, USA). The membrane was blocked with 5% non-fat milk for 1 h and incubated overnight at 4 °C with the following primary antibodies: anti-AXL (1:1000, Abcam, UK), anti-phospho-AXL (p-AXL, 1:1000, Cell Signaling Technology, USA), anti-Rac1 (1:1000, Cytoskeleton, USA), anti-phospho-Rac1 (p-Rac1, 1:1000, Abcam, UK), anti-NF-κB (1:1000, Cell Signaling Technology, USA), and anti-iNOS (1:1000, Abcam, UK). The next day, the membrane was incubated with an HRP-conjugated secondary antibody (1:5000, Sigma-Aldrich, USA) at room temperature for 1 h. Protein bands were visualized using ECL chemiluminescent substrate (Thermo Fisher Scientific, USA), and band intensity was quantified with ImageJ software, normalized to the internal control β-actin (1:1000, Sigma-Aldrich, USA). For Full and uncropped western blots, please refer to the Supplemental Material file.

### Tissue sampling and processing

After completing behavioral testing (on the ninth day post-CLP surgery), the mice were euthanized, and brain tissues, including the hippocampus and cortical regions, were rapidly collected and immediately frozen in liquid nitrogen, then stored at –80 °C for subsequent RNA extraction and sequencing. Additionally, microglia were isolated from the brains of SAE mice using flow cytometry or magnetic bead sorting. The isolated cells were used for RNA extraction and RNA-Seq analysis to investigate gene expression further changes related to the study.

### RNA extraction and quality assessment

Total RNA was extracted from treated microglia using TRIzol reagent (Thermo Fisher Scientific, USA), following the manufacturer’s instructions. RNA concentration and purity were assessed with a NanoDrop 2000 spectrophotometer (Thermo Fisher Scientific, USA), ensuring an OD260/280 ratio between 1.8 and 2.0. RNA integrity was evaluated using an Agilent 2100 Bioanalyzer (Agilent Technologies, USA), with samples requiring an RNA integrity number (RIN) of ≥7.0 to be deemed suitable for library construction and sequencing.

### RNA-Seq library construction and sequencing

To prepare for RNA-Seq sequencing, RNA libraries were constructed using the Illumina TruSeq RNA Library Preparation Kit (Illumina, USA). For each sample, 2 µg of total RNA was used, and mRNA was enriched with oligo(dT) magnetic beads. The enriched mRNA was then reverse-transcribed into cDNA using random primers, followed by adapter ligation and PCR amplification to produce the final library. Sequencing was performed on an Illumina NovaSeq 6000 platform, generating 150 bp paired-end reads with a sequencing depth of approximately 20 million reads per sample. Sequencing data were accessed via the Illumina BaseSpace service.

### Differential gene expression analysis

Sequencing data were first quality-checked to identify differentially expressed genes associated with the microglial efferocytosis and the GAS6/AXL signaling pathway using FastQC (v0.11.9). High-quality reads were aligned to the mouse genome (GRCm38) using STAR (v2.7.9a), and read counts for each gene were calculated with HTSeq (v0.11.3). Differential expression analysis was conducted using DESeq2 (v1.30.1), with low-expression genes filtered out to clean the dataset. Linear models were fitted with the limma package to compare gene expression differences between the rGAS6 and Vehicle groups. Significant differentially expressed genes were identified using the false discovery rate (FDR) correction method, and genes were categorized as upregulated or downregulated to clarify expression changes between the two groups. The selection criteria were |log_2_FC | > 1 and *p* < 0.05.

### Gene ontology (GO) and kyoto encyclopedia of genes and genomes (KEGG) functional enrichment analysis

To further understand the biological significance of the differentially expressed genes, GO and KEGG functional enrichment analyses were performed using ClusterProfiler (v3.18.1). The list of differentially expressed genes was used for enrichment analysis across GO categories (biological processes, cellular components, and molecular functions) and KEGG signaling pathways, with significant enrichment identified by *p* < 0.05 (Benjamini-Hochberg correction).

### Prediction and analysis of transcription factor binding sites

Using the JASPAR database (https://jaspar.elixir.no/), transcription factor binding sites were predicted to determine the enrichment of binding sites in the regulatory regions upstream of genes associated with anti-inflammatory and efferocytosis functions, as well as pro-inflammatory genes. This binding site enrichment indicates the potential roles of specific transcription factors in regulating the expression of these genes.

### Primary microglia culture and treatment

To investigate the effects of GAS6 on microglial function, primary microglia were isolated and cultured from mice in the Sham, SAE, rGAS6, rGAS6 + R428, and rGAS6 + NSC23766 treatment groups. A finely drawn glass capillary was used, under the aid of a microscope, to gently puncture the transparent membrane of the retrocerebellar cisterna. Care was taken to avoid damaging blood vessels to prevent blood contamination of the cerebrospinal fluid. After a puncture, cerebrospinal fluid was slowly aspirated using capillary action or a micropipette. Twenty-four 1- to 2-day-old C57BL/6 N mice (purchased from Home-SPF (Beijing) Biotechnology Co., Ltd.) were anesthetized with isoflurane, and brains were quickly extracted. The cerebral cortex was isolated on ice and digested with 0.05% trypsin/EDTA at 37 °C for 15 min. Digestion was terminated by adding DMEM supplemented with 10% FBS (Thermo Fisher Scientific, USA). After centrifugation at 300 × *g* for 5 min, cells were plated on poly D-lysine-coated dishes (MilliporeSigma, USA) and cultured in DMEM containing 10% FBS and B27 (Thermo Fisher Scientific, USA) at 37 °C in a 5% CO_2_ incubator. On day 10, microglia were collected by shaking the culture flasks at 5 × g for 2 h and then seeded into dishes for subsequent experiments.

### Primary cortical neuron culture and induction of in vitro ischemia

Mice were rapidly dissected, and their cortical tissue was isolated and digested with 0.05% EDTA. Cells were then seeded on poly-D-lysine-coated culture dishes (MilliporeSigma, USA) and cultured in a Neurobasal medium supplemented with B27 (Thermo Fisher Scientific, USA). On day 10, neurons were subjected to 3 h of oxygen-glucose deprivation (OGD) to establish an in vitro ischemia model.

### Study of efferocytosis in neurons and microglia

Primary cultured microglia were plated on poly-D-lysine-coated coverslips in a 24-well plate (2 × 10^5^ cells/well) and cultured for 24 h. Propidium iodide (PI)-stained dead or dying neurons were then added to the microglia at a ratio of 10:1 and incubated for 1 to 6 h in a DMEM medium containing 2% FBS (Thermo Fisher Scientific, USA). After incubation, cells were washed with PBS (Thermo Fisher Scientific, USA) and fixed with 4% paraformaldehyde. Microglia were stained for F-actin with anti-Iba1 antibody (Wako Chemicals, USA) and Alexa Fluor 488-conjugated phalloidin (A12379, 1:300 in PBS; Invitrogen, USA) and incubated at room temperature for 1 h in the dark. Images were captured using a confocal microscope.

To analyze the roles of STAT3 and SP1 in apoptotic cell clearance, microglia in the rGAS6 treatment group were pre-treated with specific inhibitors: STAT3 inhibitor Stattic (100 μM, MedChemExpress, USA) and SP1 inhibitor Plicamycin (100 μM, MedChemExpress, USA) for 1 h. After washing, cells were subjected to an apoptotic cell clearance assay. Images were processed with ImageJ software, automatically identifying and counting cells unbiasedly.

### Chromatin immunoprecipitation (ChIP) assay

A ChIP assay was performed to verify whether STAT3 and SP1 bind to the promoter regions of target genes (Arg1, CD206, MerTK, and TREM2) and regulate their expression. Microglia isolated from the cerebral cortex were fixed with 1% formaldehyde to stabilize protein-DNA interactions, and the reaction was quenched with 125 mM glycine, followed by two PBS washes. The cells were then lysed, and chromatin was sonicated to produce fragments between 200 and 1000 bp. The sheared chromatin was incubated overnight with either anti-STAT3 (1:100) or anti-SP1 (1:100) antibodies on protein A/G magnetic beads, with an IgG control group for comparison. The beads were washed with buffer the next day, and DNA was eluted. After reversing the cross-links, qPCR extracted and analyzed DNA to assess the enrichment of target gene promoter regions (Arg1, CD206, MerTK, and TREM2). Relative enrichment was calculated using the ΔΔCt method, with primer sequences listed in Table S1.

### Lentiviral production and cell transfection

Based on GenBank, potential short hairpin RNA (shRNA) target sequences were analyzed and designed to target AXL and Rac1. Oligonucleotides were synthesized by GenePharma® (Shanghai, China). A lentiviral packaging system was constructed using the pLKO.1 vector (lentiviral gene silencing vector). Packaging plasmids and target constructs were co-transfected into HEK293T cells (80–90% confluency) using Lipofectamine 3000 (Thermo Fisher Scientific, USA). After 48 h of culture, the supernatant containing viral particles was collected, filtered through a 0.22 μm membrane, and centrifuged. The viral supernatant was mixed with Lenti-X™ Concentrator (Takara, Japan) at a ratio of 3:1, then gently inverted to mix and incubated at 4 °C overnight. Following centrifugation at 5000 × *g* for 30 min, the supernatant was discarded, and the viral pellet was resuspended in 100 μL of DMEM by gently pipetting at the bottom of the tube.

At approximately 80% confluence, 100 μL of viral solution and 10 mg/μL polybrene were added in microglia. After 6 h, 500 μL of complete DMEM (10% FBS and 1% streptomycin-penicillin) was added, and cells were cultured overnight. Cells were then collected by centrifugation, the supernatant was discarded, and 1 mL of fresh complete DMEM was added. Stable cell lines were selected using 2 μg/mL puromycin (Sigma-Aldrich, Germany) for two weeks [33].

Cells were divided into the following transfection groups: (1) sh-NC group, transduced with lentivirus containing the negative control knockdown vector; (2) sh-AXL group, transduced with lentivirus containing the sh-AXL knockdown vector; and (3) sh-Rac1 group, transduced with lentivirus containing the sh-Rac1 knockdown vector. Forty-eight hours post-transduction, RT-qPCR was performed to verify mRNA knockdown and overexpression efficiency. All plasmids were designed and synthesized by RiboBio (Guangzhou, China).

Primer sequences were as follows: negative control (NC), 5ʹ-UUC UCC GAA CGU GUC ACG U-3ʹ; sh-AXL, 5ʹ-CTTTAGGTTCTTTGCTGCATT-3ʹ; sh-Rac1, 5ʹ-CGCAAACAGATGTGTTCTTAA-3ʹ.

### Data analysis

All experiments were performed in triplicate, and data are presented as mean ± SEM deviation. Statistical analysis was conducted using GraphPad Prism 8 software (GraphPad Software, USA). Differences between groups were evaluated by one-way analysis of variance (ANOVA), with *p* values < 0.05 considered statistically significant.

## Supplementary information

Original Data(Full length western blots)

Supplemental Material

## Data Availability

All data can be provided as needed.
