# Correction: GAS6/AXL signaling promotes M2 microglia efferocytosis to alleviate neuroinflammation in sepsis-associated encephalopathy

**DOI:** 10.1038/s41420-025-02706-3

**Published:** 2025-11-17

**Authors:** Yuedong Tang, Hanbing Hu, Qiliang Xie, Jie Shen

**Affiliations:** 1 Center of Emergency and Critical Medicine, Jinshan Hospital of Fudan University, Shanghai, 201508 China; 2 Shanghai Institute of Infectious Disease and Biosecurity, Fudan University, Shanghai, 200032 China; 3 Research Center for Chemical Injury, Emergency and Critical Medicine of Fudan University, Shanghai, 201508 China; 4 Jinshan Hospital of Fudan University, Key Laboratory of Chemical Injury, Emergency and Critical Medicine of Shanghai Municipal Health Commission, Shanghai, 201508 China

**Keywords:** Molecular biology, Diseases

Correction to: *Cell Death Discovery* 10.1038/s41420-025-02507-8, published online 06 June 2025

In this article the figure 1, 2 4 and 6 have not been replaced with the updated version during the correction process.
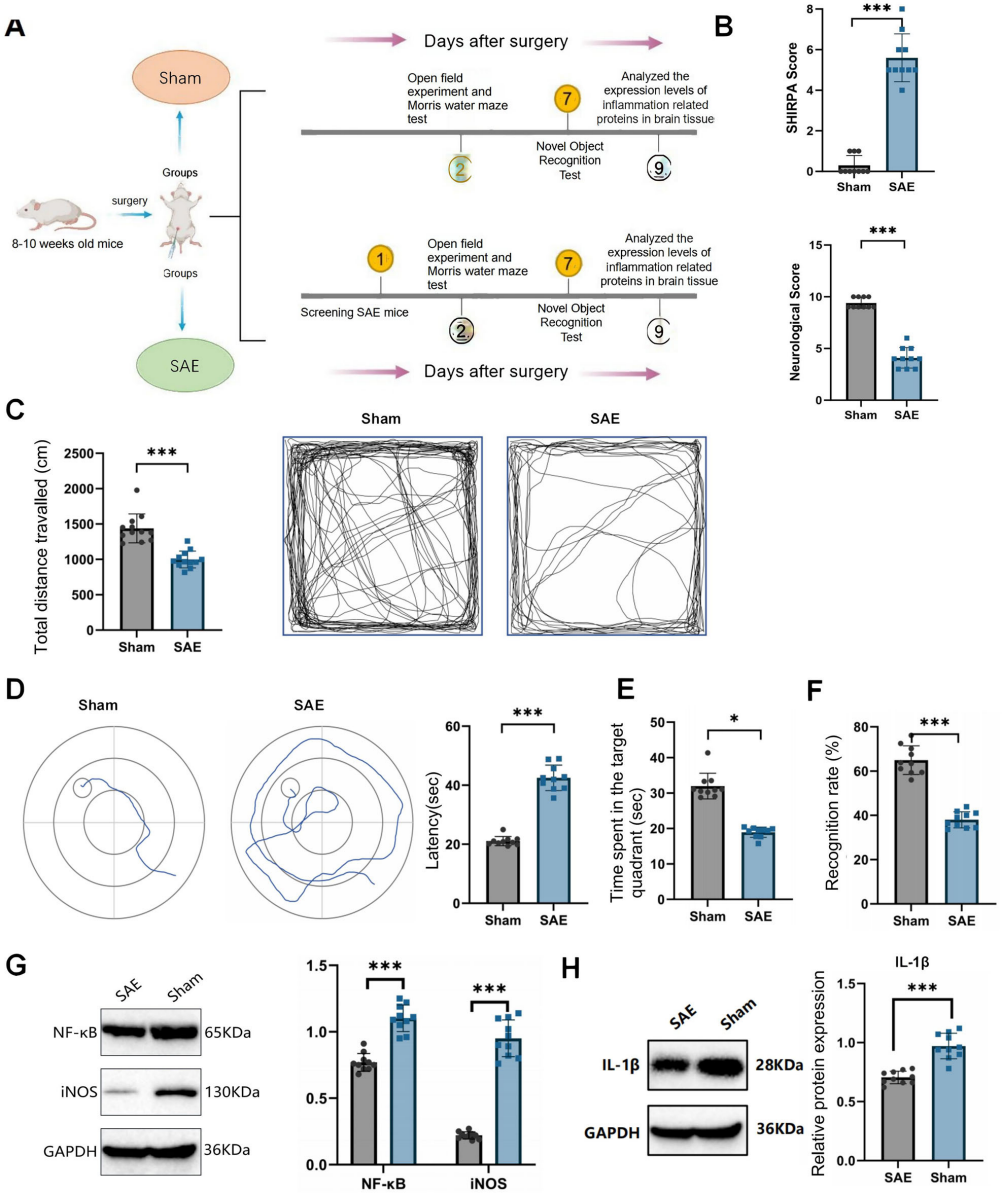

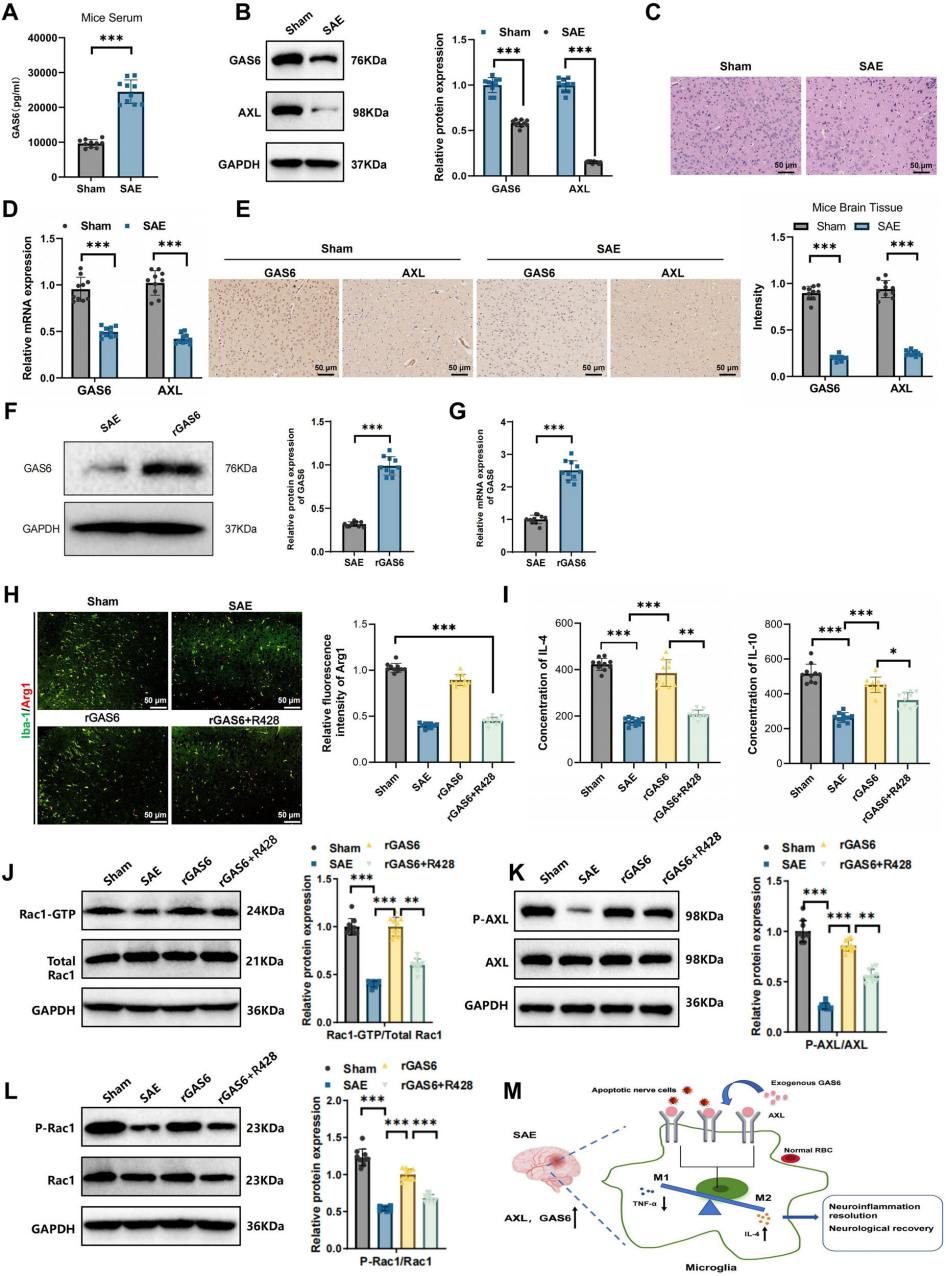

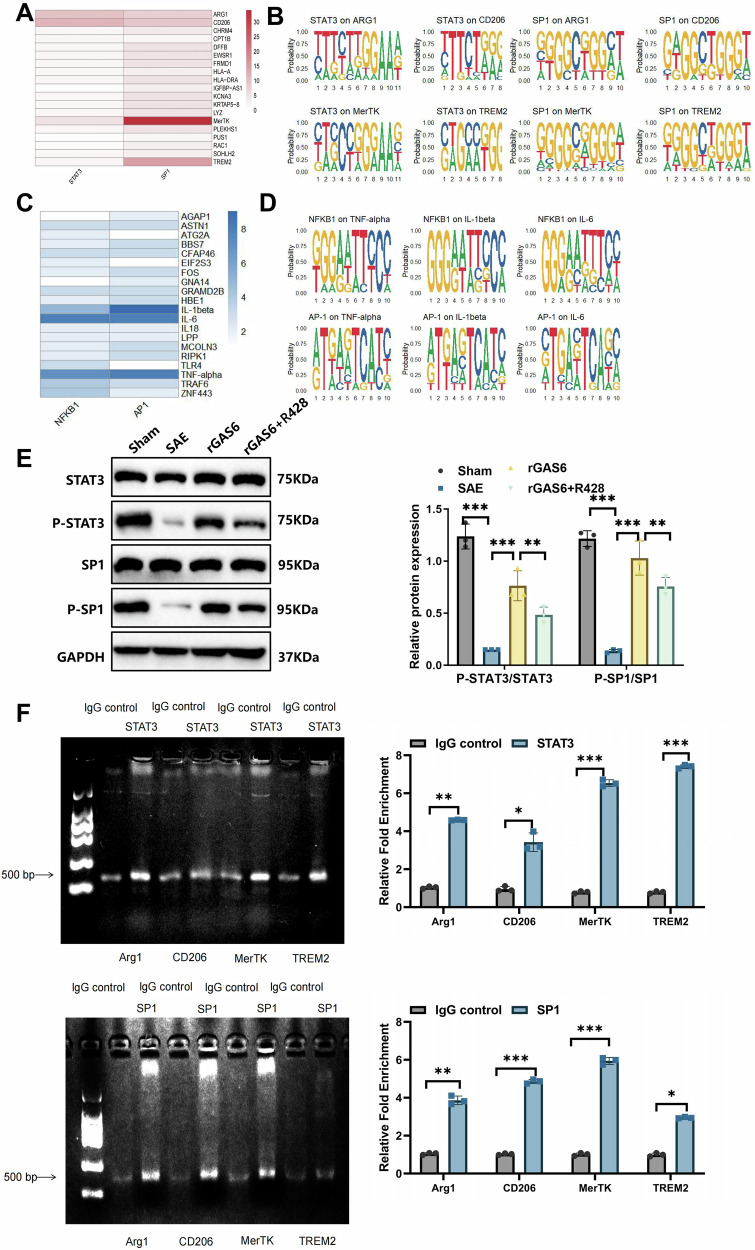

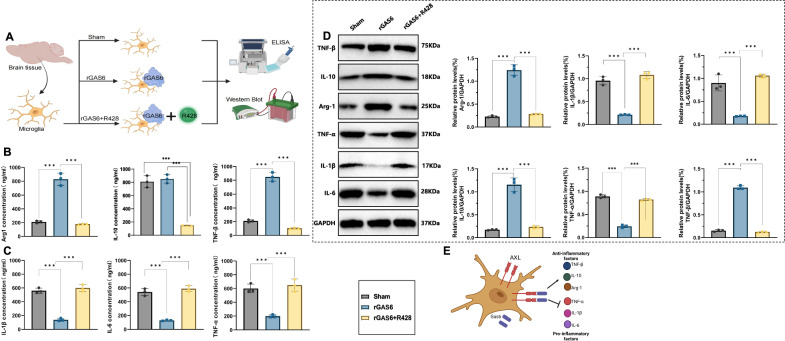


The original article has been corrected.

