# Supplementary material for: GAS6/AXL signaling promotes M2 microglia efferocytosis to alleviate neuroinflammation in sepsis-associated encephalopathy
Source: Cell Death Discov. 2025 Jun 6;11:268. doi: 10.1038/s41420-025-02507-8 (PMC12144116; doi:10.1038/s41420-025-02507-8)
Supplement: Supplementary file 1 — Original Data(Full length western blots) [file 41420_2025_2507_MOESM1_ESM.docx]

**Full and uncropped western blots**


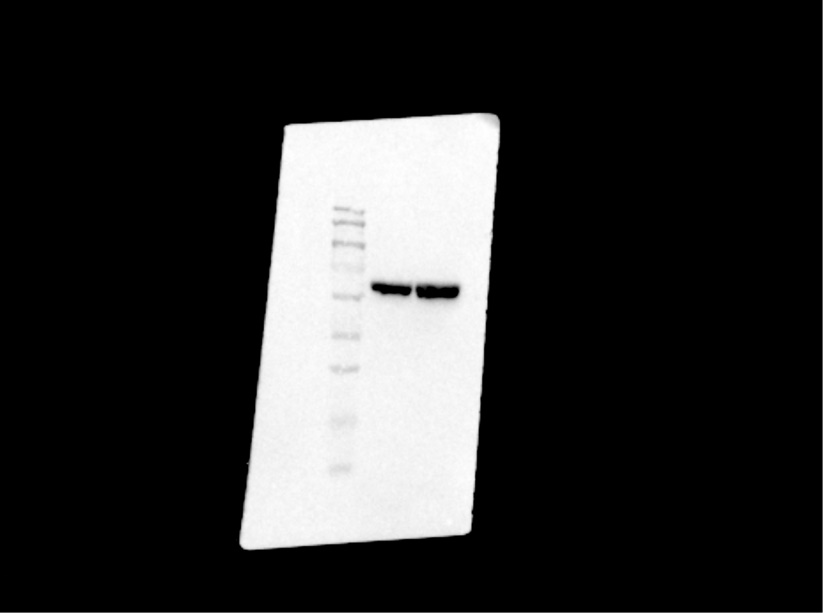


Full and uncropped western blots for Figure 1G-1


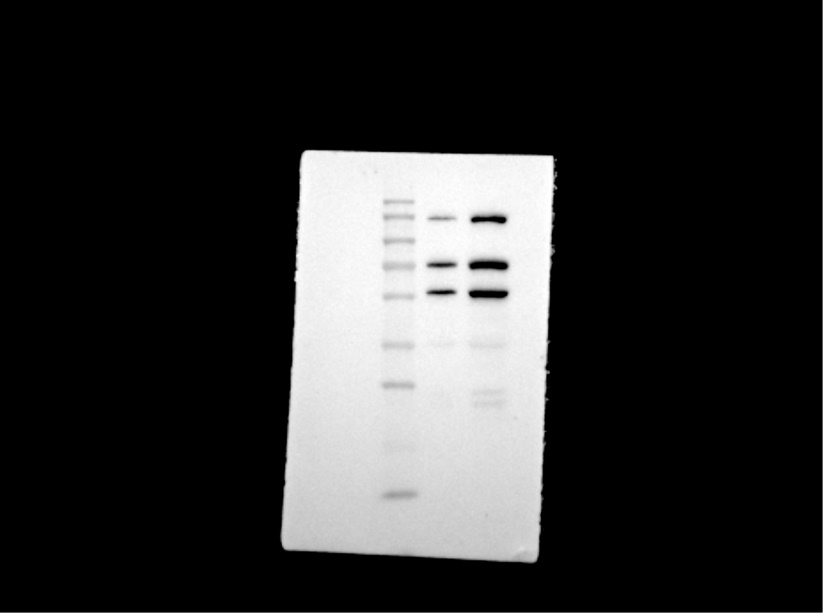


Full and uncropped western blots for Figure 1G-2


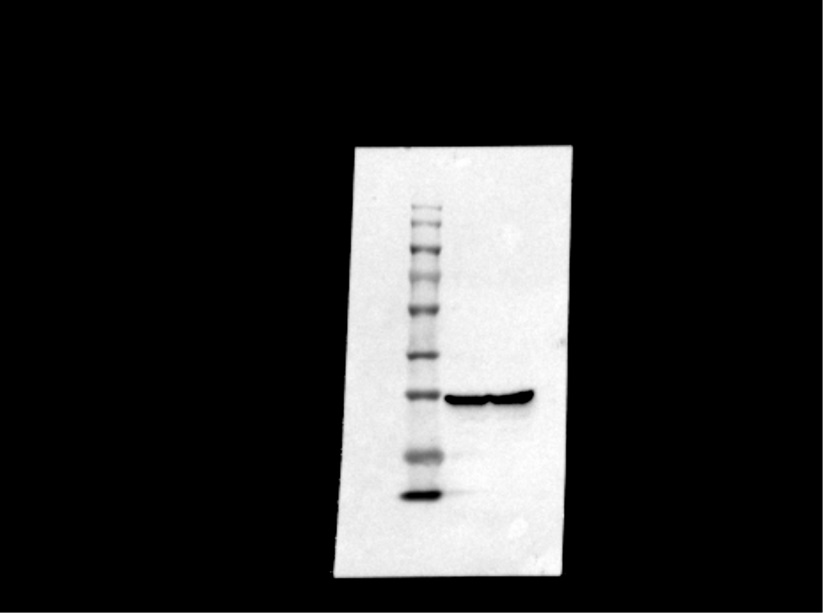


Full and uncropped western blots for Figure 1G-3


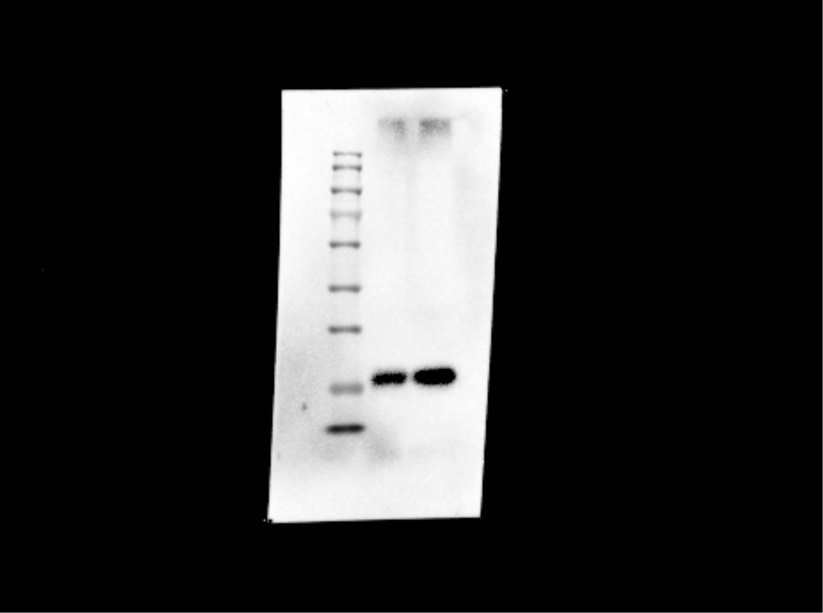


Full and uncropped western blots for Figure 1H-1


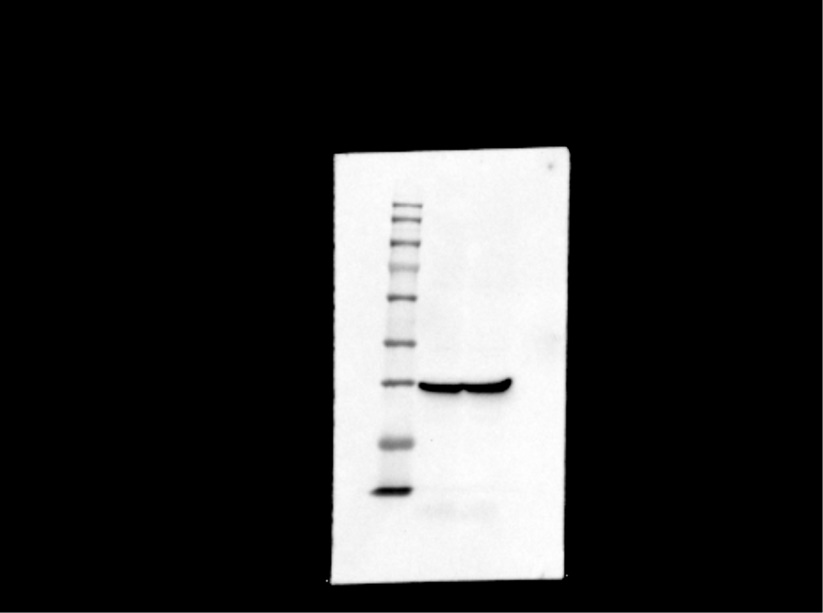


Full and uncropped western blots for Figure 1H-2


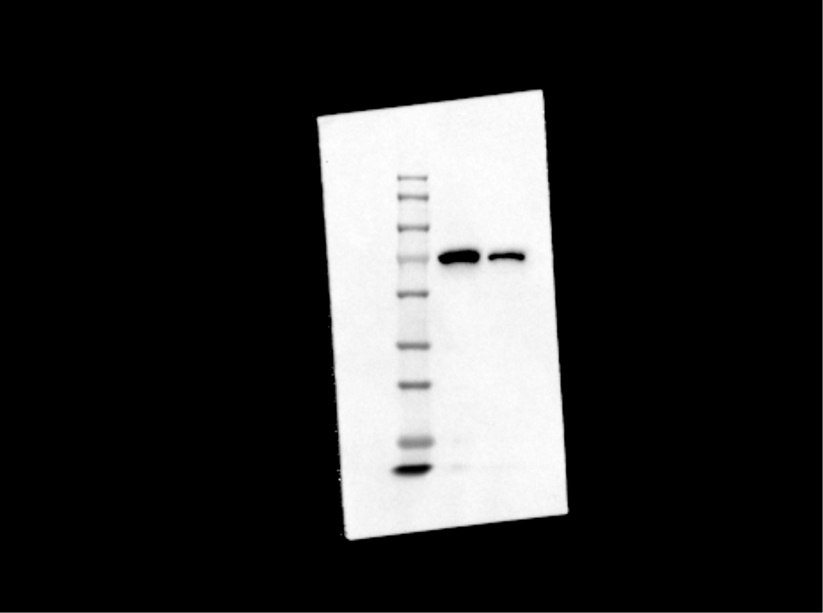


Full and uncropped western blots for Figure 2B-1


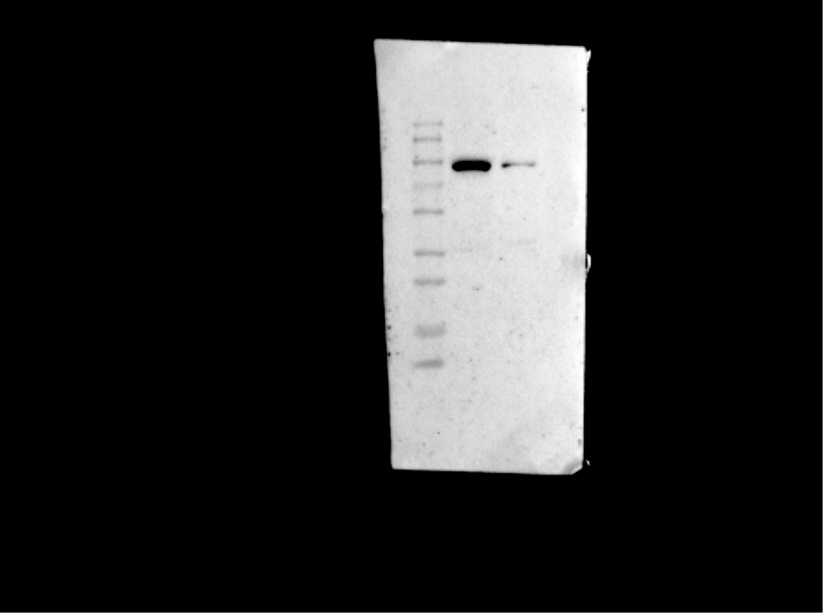


Full and uncropped western blots for Figure 2B-2


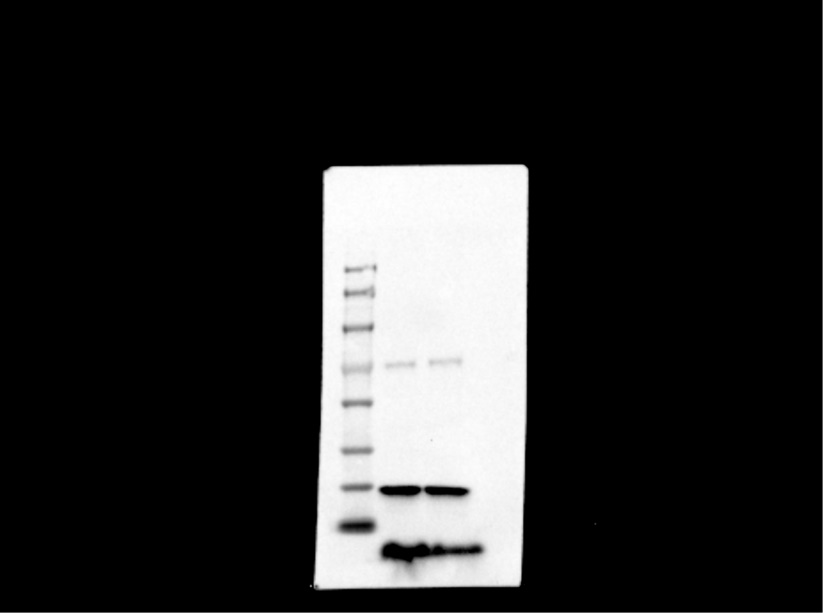


Full and uncropped western blots for Figure 2B-3


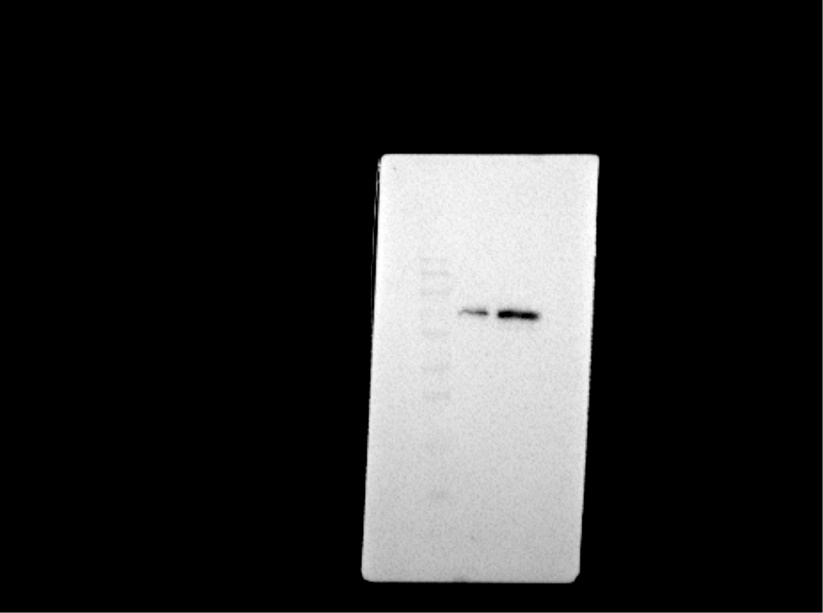


Full and uncropped western blots for Figure 2F-1


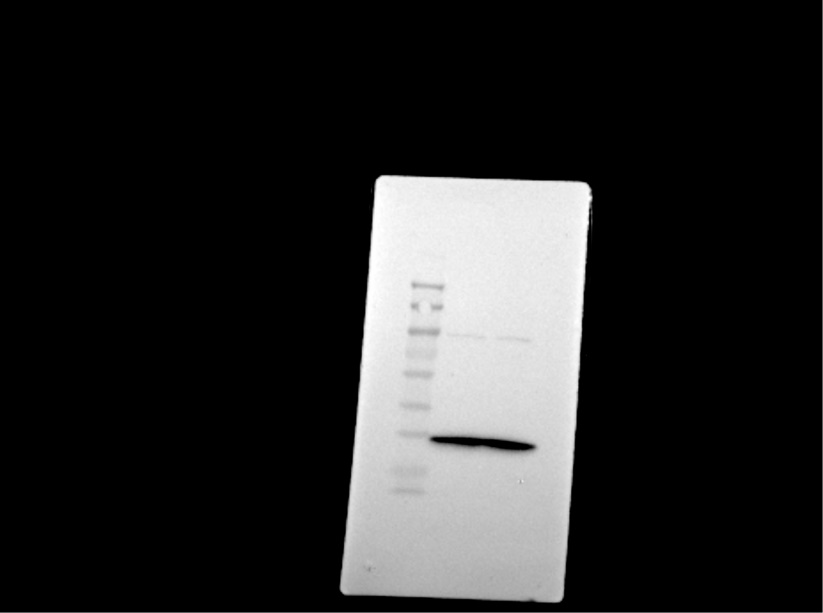


Full and uncropped western blots for Figure 2F-2


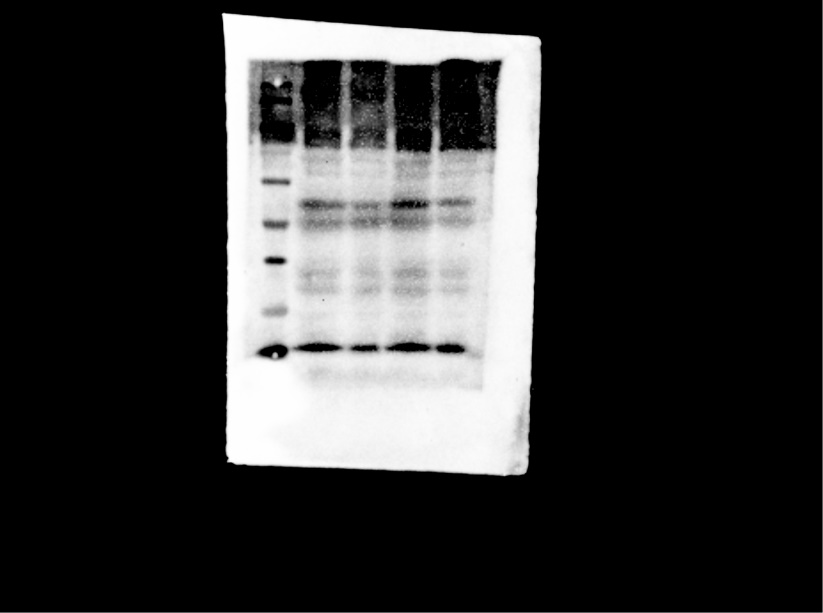


Full and uncropped western blots for Figure 2J-1


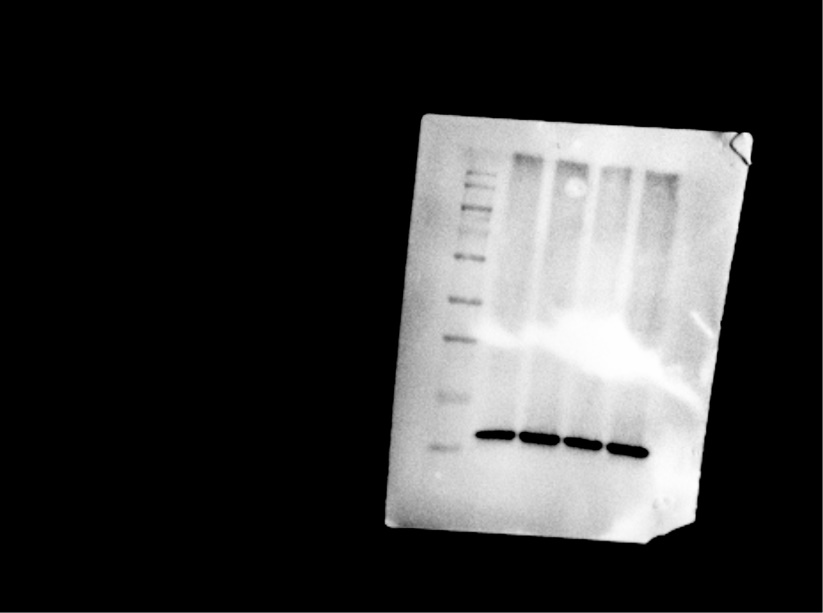


Full and uncropped western blots for Figure 2J-2


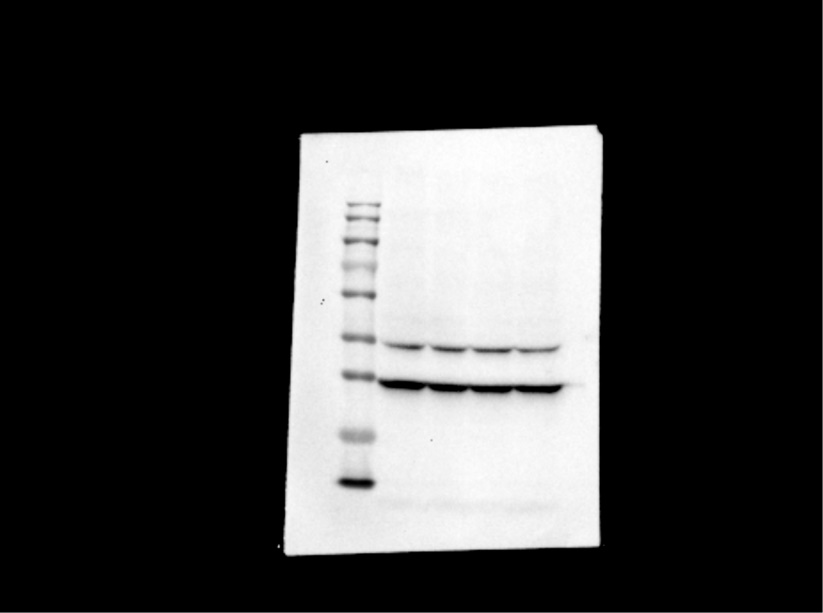


Full and uncropped western blots for Figure 2J-3


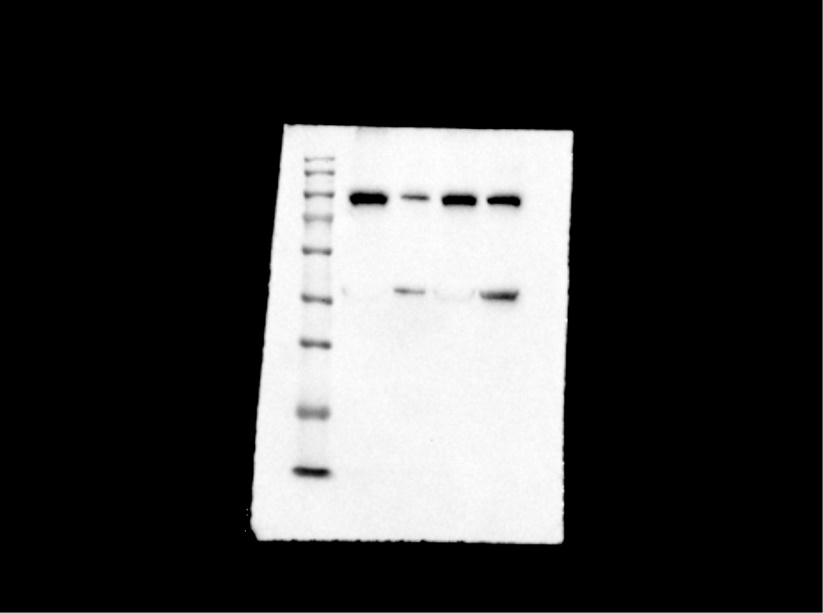


Full and uncropped western blots for Figure 2K-1


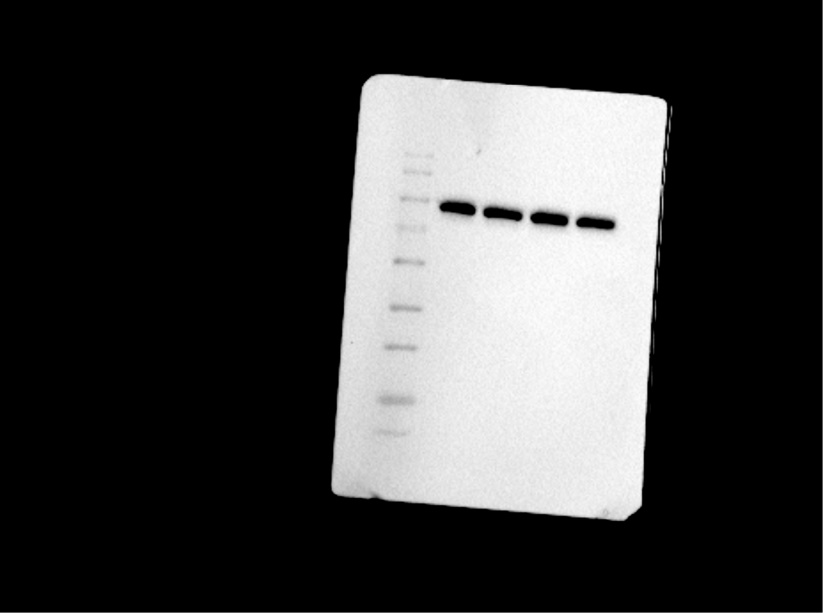


Full and uncropped western blots for Figure 2K-2


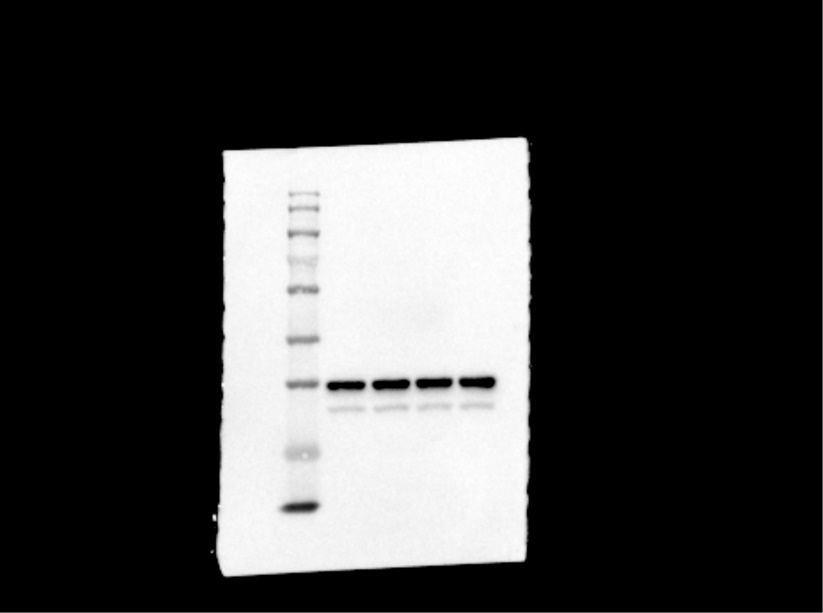


Full and uncropped western blots for Figure 2K-3


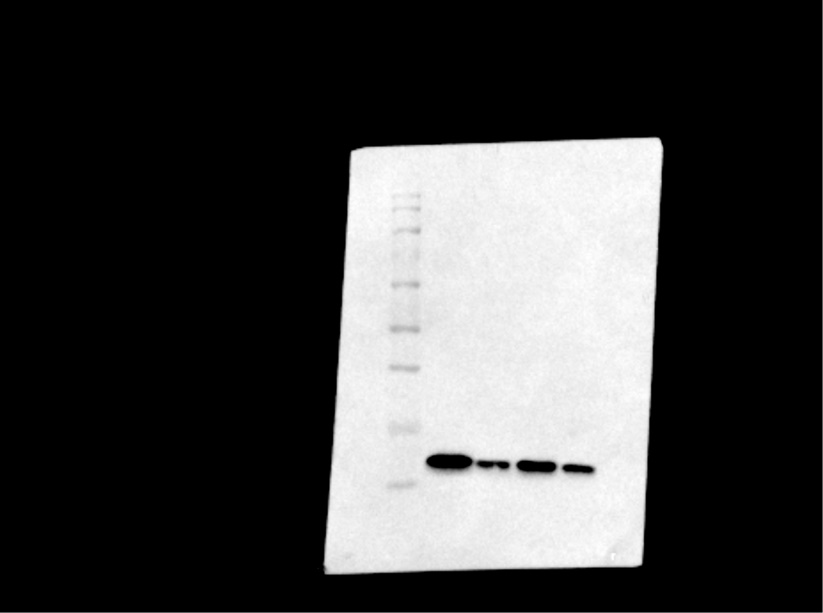


Full and uncropped western blots for Figure 2L-1


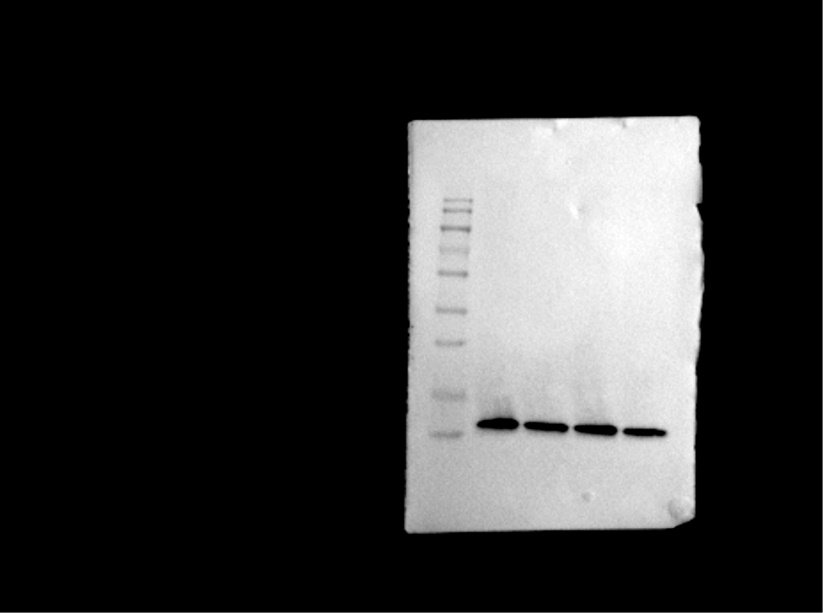


Full and uncropped western blots for Figure 2L-2


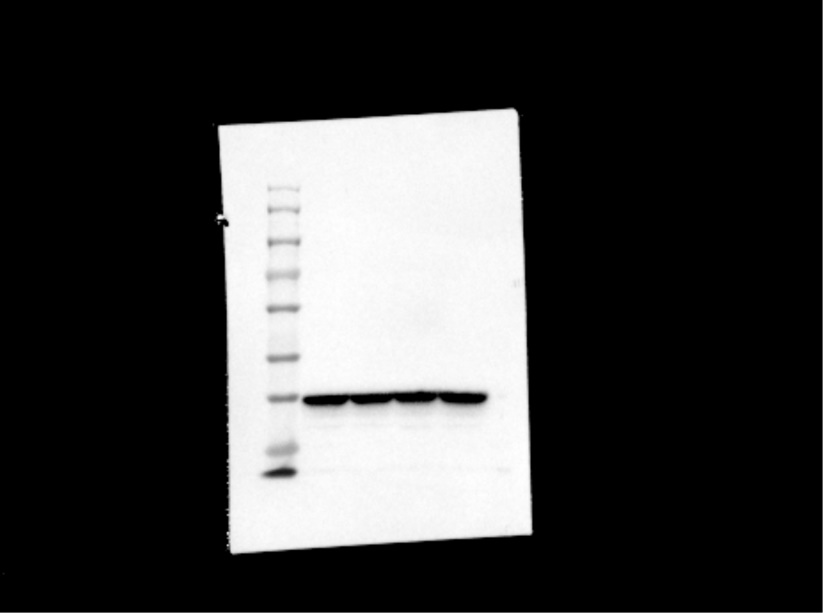


Full and uncropped western blots for Figure 2L-3


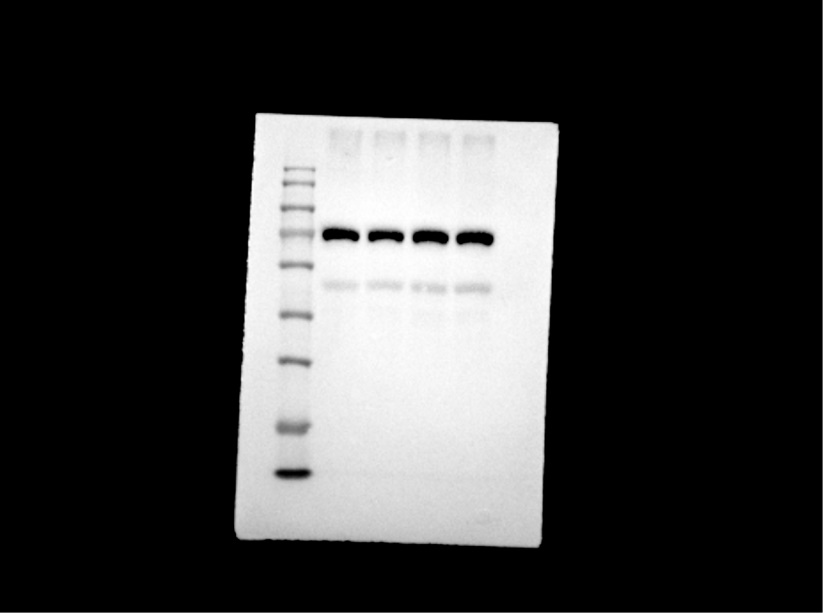


Full and uncropped western blots for Figure 4E-1


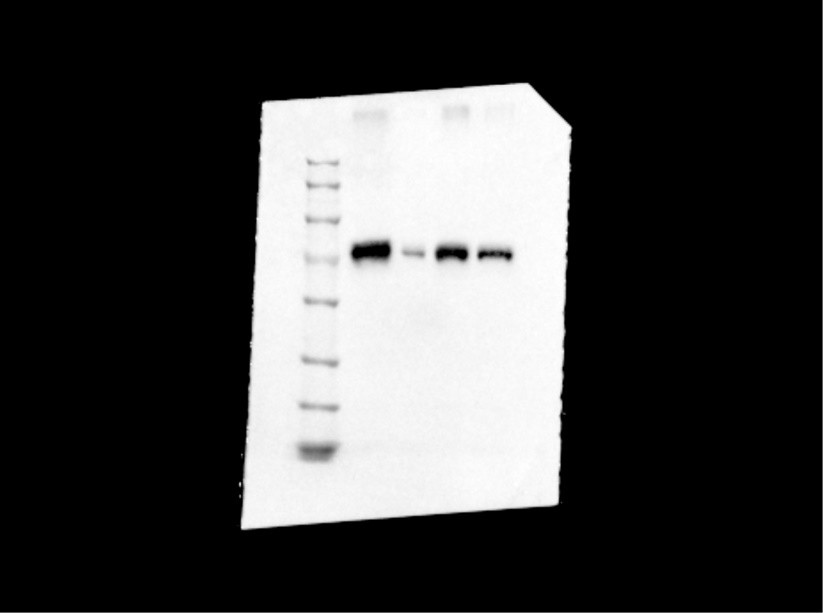


Full and uncropped western blots for Figure 4E-2


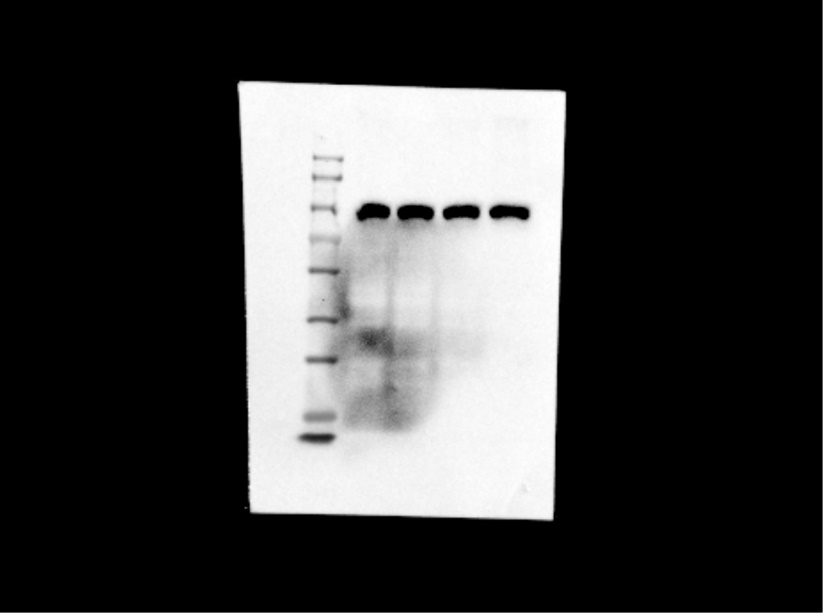


Full and uncropped western blots for Figure 4E-3


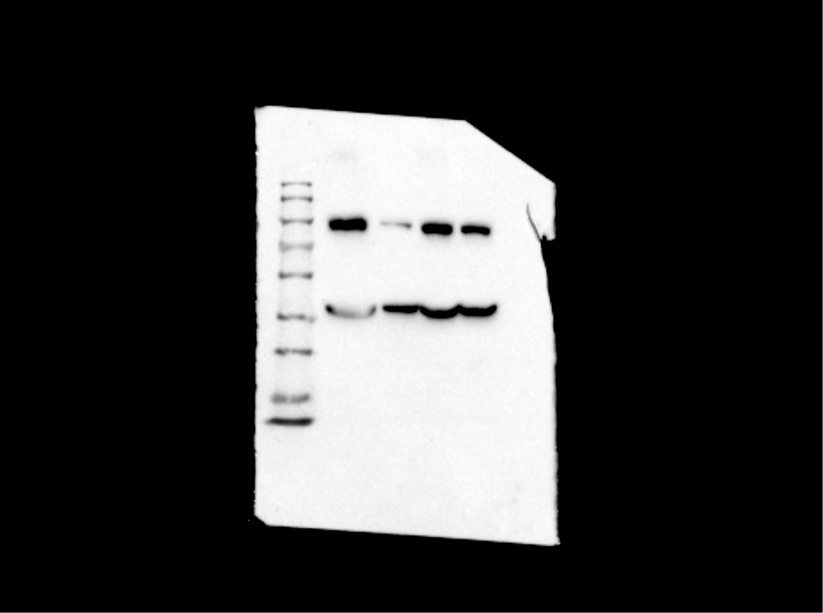


Full and uncropped western blots for Figure 4E-4


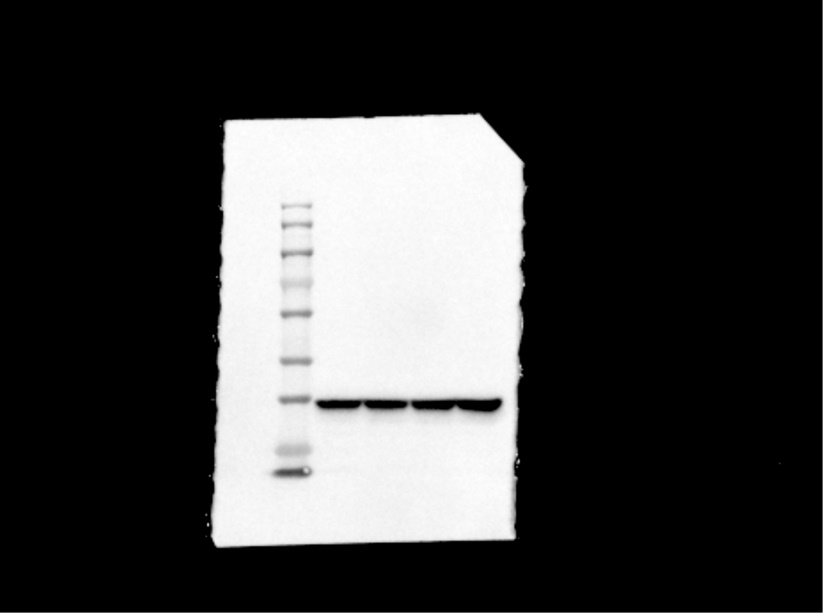


Full and uncropped western blots for Figure 4E-5


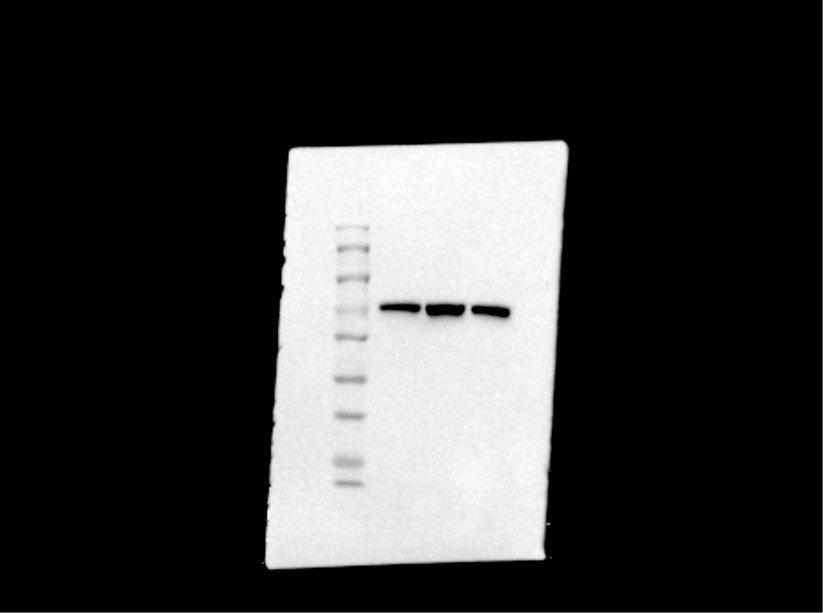


Full and uncropped western blots for Figure 6D-1


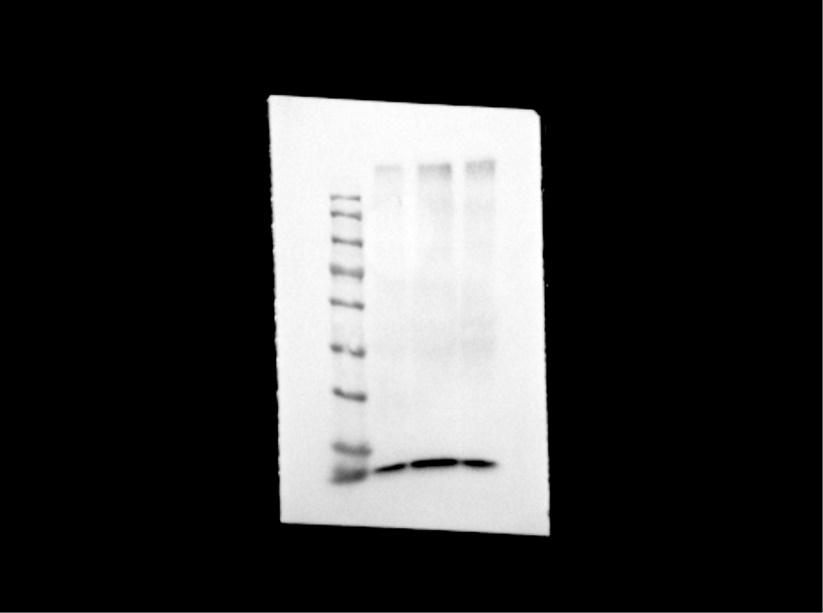


Full and uncropped western blots for Figure 6D-2


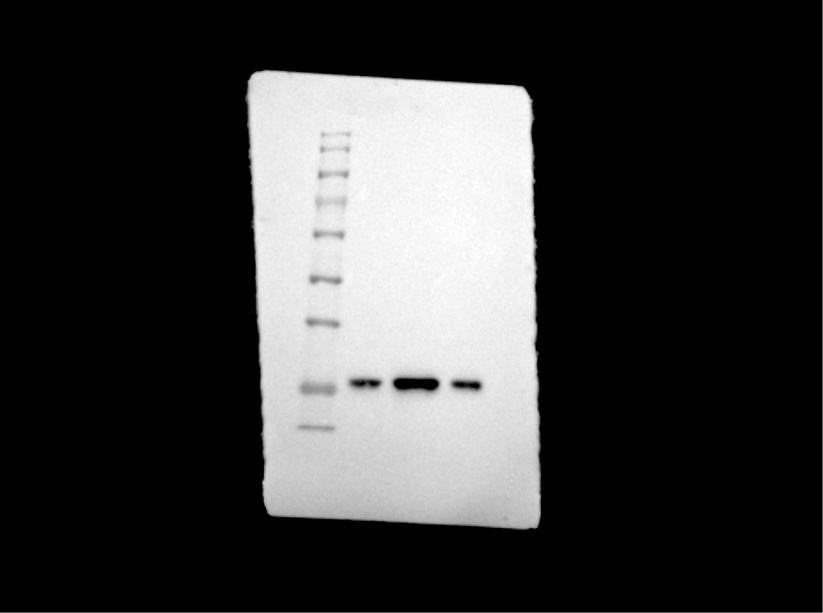


Full and uncropped western blots for Figure 6D-3


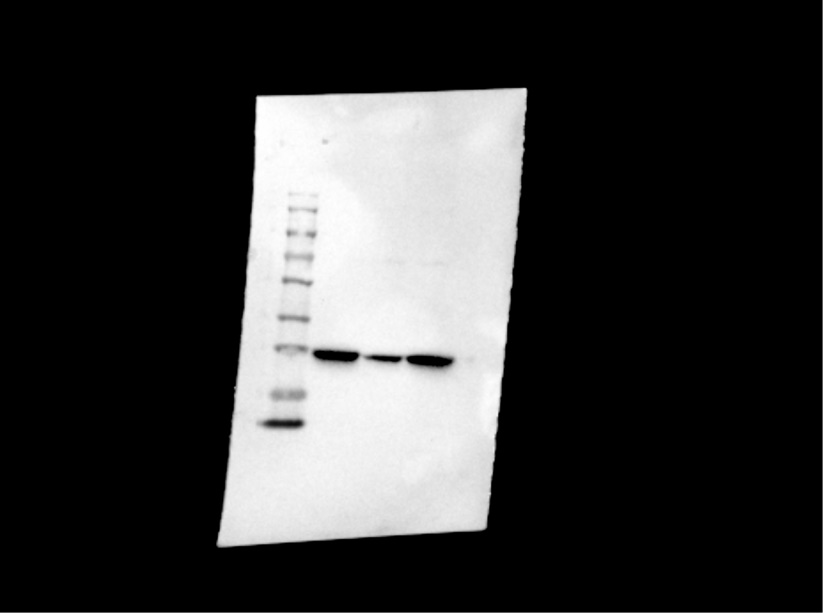


Full and uncropped western blots for Figure 6D-4


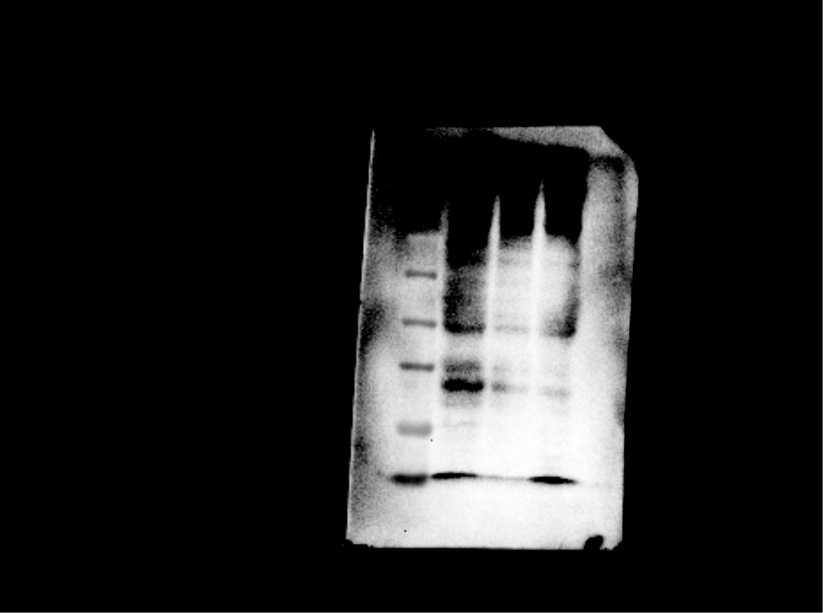


Full and uncropped western blots for Figure 6D-5


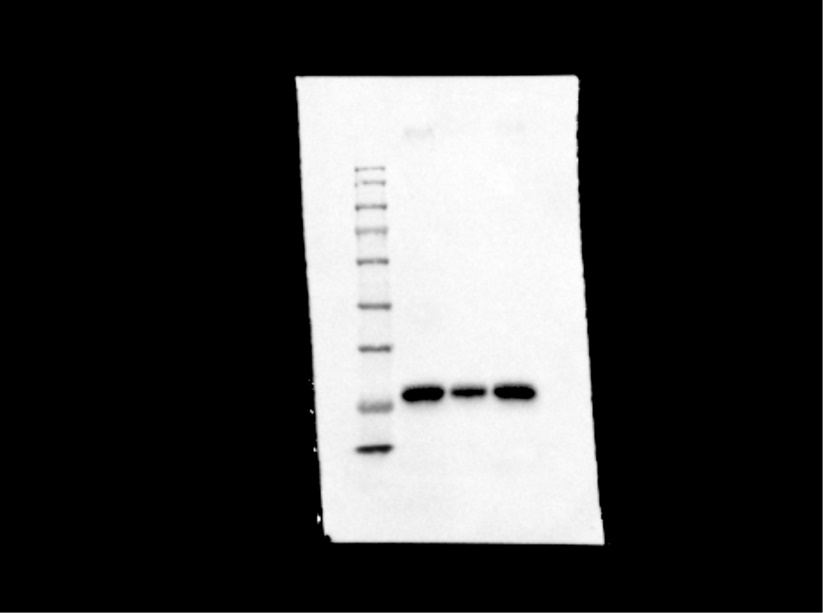


Full and uncropped western blots for Figure 6D-6


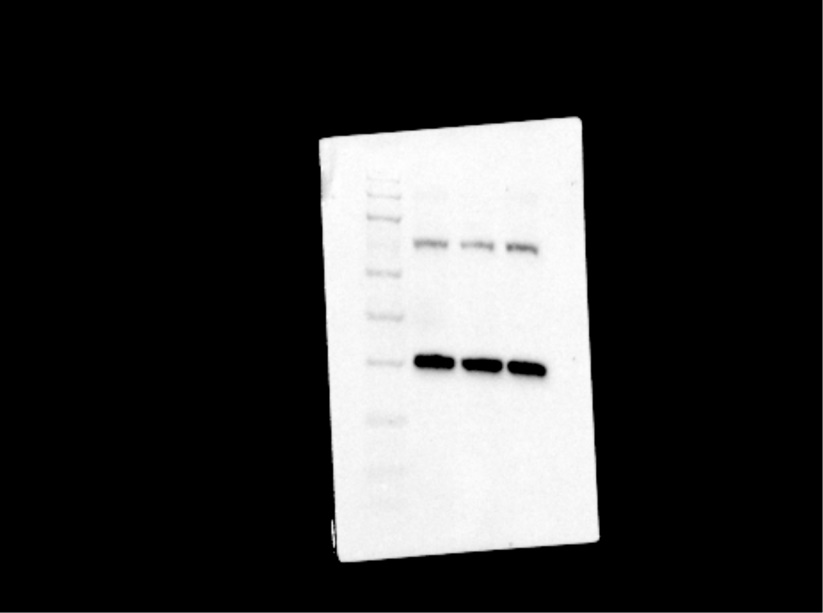


Full and uncropped western blots for Figure 6D-7


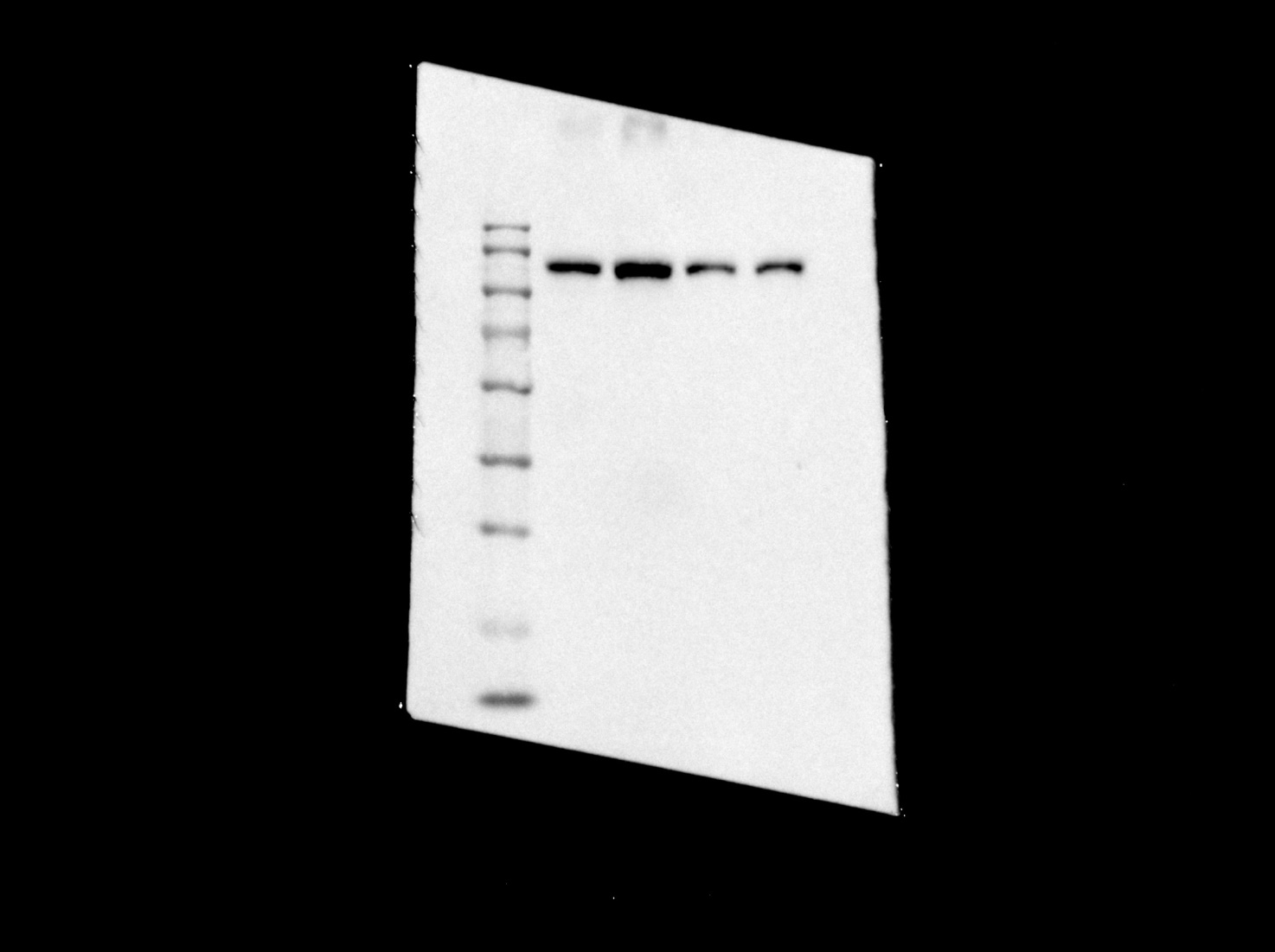


Full and uncropped western blots for Figure 7B-1


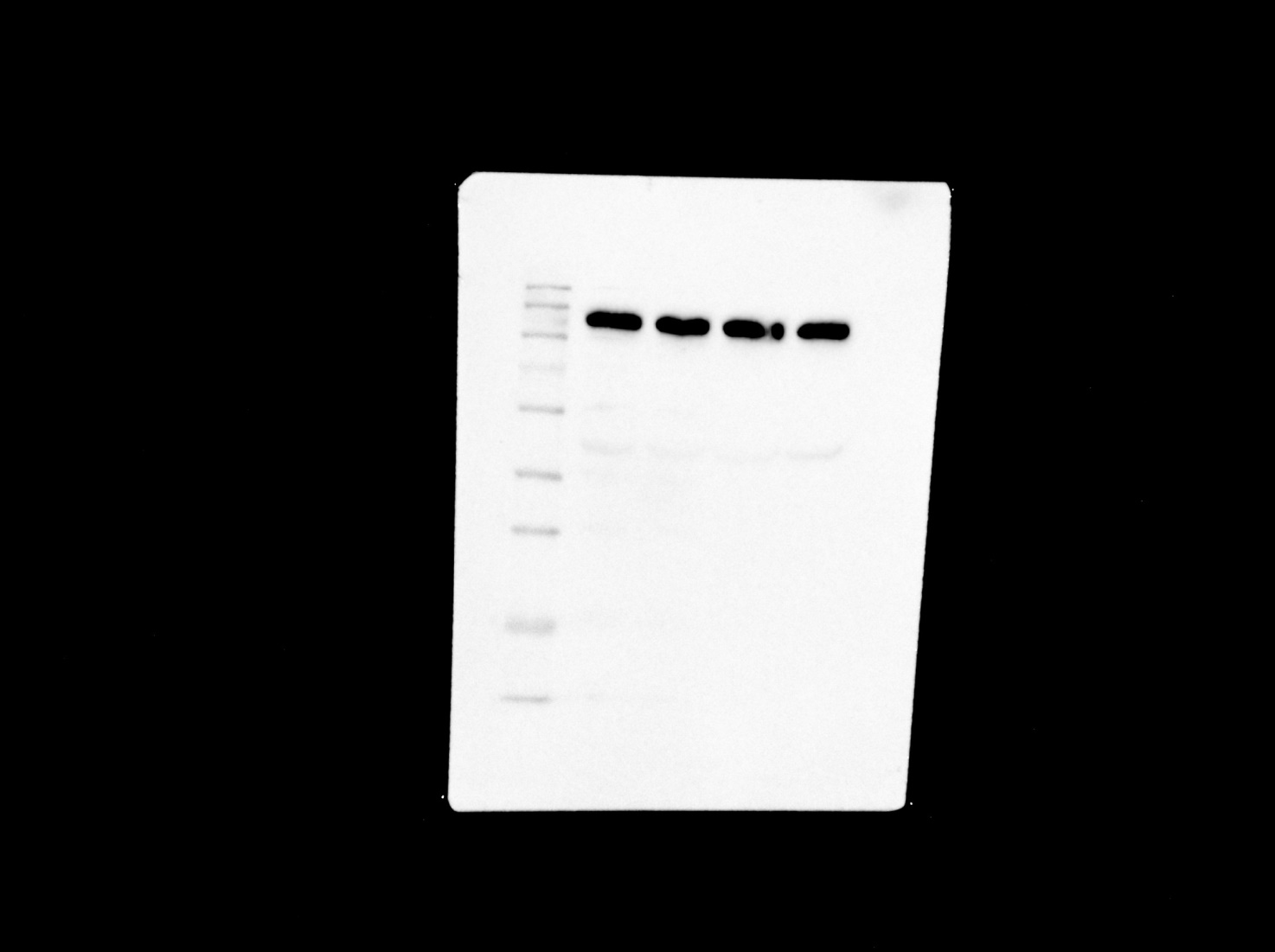


Full and uncropped western blots for Figure 7B-2


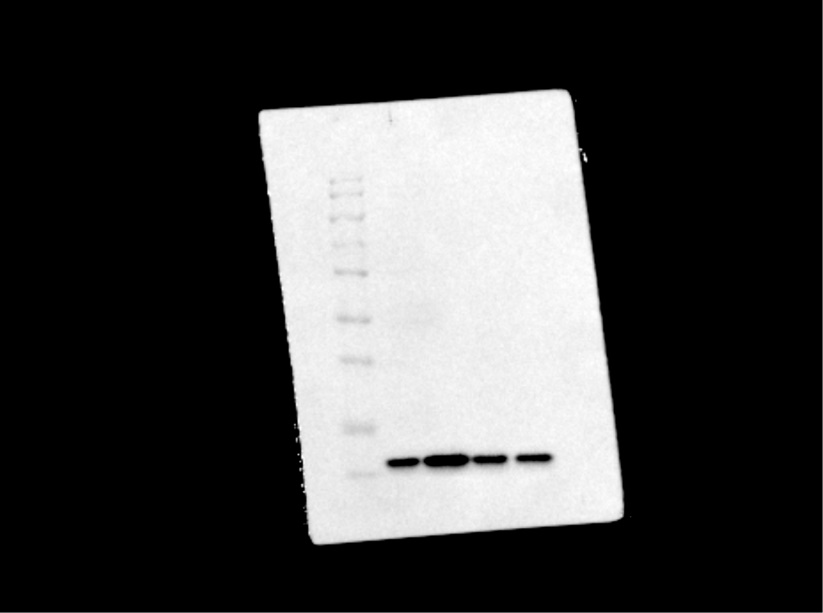


Full and uncropped western blots for Figure 7B-3


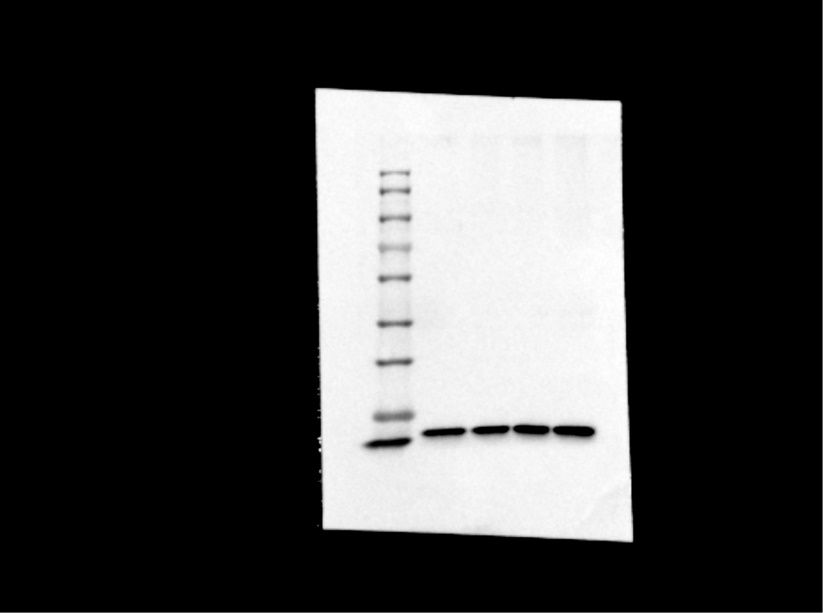


Full and uncropped western blots for Figure 7B-4


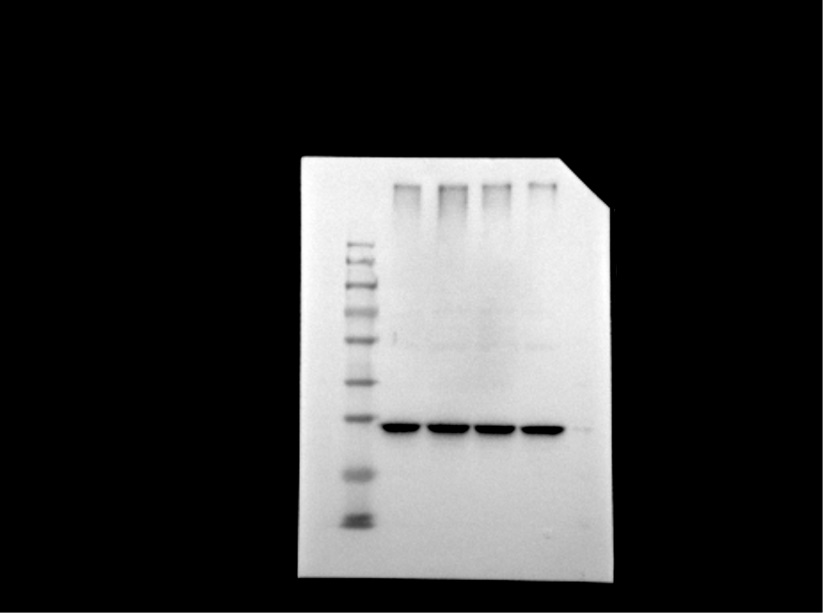


Full and uncropped western blots for Figure 7B-5
