# Supplementary material for: GAS6/AXL signaling promotes M2 microglia efferocytosis to alleviate neuroinflammation in sepsis-associated encephalopathy
Source: Cell Death Discov. 2025 Jun 6;11:268. doi: 10.1038/s41420-025-02507-8 (PMC12144116; doi:10.1038/s41420-025-02507-8)
Supplement: Supplementary file 2 — Supplemental Material [file 41420_2025_2507_MOESM2_ESM.pdf]

Table S1. Gene and primer sequence.

Gene	Forward Primer	Reverse Primer
Arg1	AAGATTCCCGATGTGCCAGG	GTCCACGTCTCTCAAGCCAA
CD206	GCCTCGTTGTTTTGCGTCTT	GAGAACAGCACCCGGAATGA
MerTK	CCGCCCCACCTTTTCAGTAT	GAGCTCTCCAGCAACTGTGT
TREM2	ATGCTGGAGATCTCTGGTTCC	CTCAGCCCTGGAGATGCTGT
GAPDH	GACAGTCAGCCGCATCTTCT	GCGCCCAATACGACCAAATC
